# ﻿First report of the *Euconnus* Thomson subgenus *Cladoconnus* Reitter in the New World, represented by thirteen new Appalachian species (Coleoptera, Staphylinidae, Scydmaeninae)

**DOI:** 10.3897/zookeys.1137.97068

**Published:** 2022-12-22

**Authors:** Michael S. Caterino

**Affiliations:** 1 Dept of Plant & Environmental Sciences, Clemson University, Clemson, SC 29634, USA Clemson University Clemson United States of America

**Keywords:** Biodiversity, dark taxa, leaf litter, metabarcoding

## Abstract

Thirteen new species of *Euconnus* Thomson (Staphylinidae: Scydmaeninae: Glandulariini) are described from the southern Appalachian Mts, USA: *Euconnusmegalops***sp. nov.**, *E.vexillus***sp. nov.**, *E.cumberlandus***sp. nov.**, *E.vetustus***sp. nov.**, *E.adversus***sp. nov.**, *E.astrus***sp. nov.**, *E.cultellus***sp. nov.**, *E.falcatus***sp. nov.**, *E.cataloochee***sp. nov.**, *E.kilmeri***sp. nov.**, *E.draco***sp. nov.**, *E.tusquitee***sp. nov.**, and *E.attritus***sp. nov.** These share a number of morphological characters with the Old World subgenus Cladoconnus Reitter, representing a diversification of species distinct from anything previously known from the western hemisphere. Most of the species occur at higher elevations, some at the tops of the region’s highest mountains, and a few are single-peak endemics. No females of these species are winged, and in several species neither sex is winged. A preliminary phylogeny suggests the wingless species represent a clade within a clade of wing-dimorphic species.

## ﻿Introduction

Following a century of near neglect, the hyperdiverse scydmaenine (Staphylinidae) genus *Euconnus* Thomson has seen something of a resurgence of interest. With over 2500 described species, the genus ranks as one of the most species-rich genera of organisms on the planet. Pawel Jałoszyński (2012 and others) has recently attempted to bring some order to the classification of these species on a global scale, revising the types of most of the 30+ subgenera, elevating, synonymizing, or validating the species of most, motivated by and also acknowledging the fact that many hundreds of undescribed species exist, many of uncertain placement in the cumbersome and inconsistent intrageneric taxonomy.

On the North American front, Stephan et al. (2021) recently revised the ‘then’-valid subgenus Napochus, describing and redescribing more than 100 species in the group. Jałoszyński’s (2021) subgeneric revision subsequently demoted *Napochus* to synonymy with the nominate *Euconnus* s. str., finding no consistent characters to distinguish them. Synonymizing the monotypic *Pycnophus* in the same paper, following several other changes (e.g. Jałoszyński 2017) leaves *Euconnus* with only three valid subgenera in the Nearctic, *Psomophus* Casey, *Tetramelus* Motschulsky, and *Euconnus* s. str., compared with 10 subgenera recognized in American Beetles (O’Keefe 2001). Apart from those formerly contained in *Napochus*, none of these has been revised since the foundational treatment of Casey (1897), with few species even having been described in the interim.

Recent work in the higher parts of the southern Appalachian mountains of the southeastern USA revealed numerous new *Euconnus* species. This on its own was not particularly surprising, but the difficulty of assigning all of them to subgenus was. Several of these species exhibit a sexual dimorphism that turns out to be known from only a single subgenus, *Cladoconnus* Reitter, hitherto known only from the Palaearctic region (Hlaváč and Stevanović 2013; Jałoszyński 2018), the males having longitudinal carinae on the inner edges of the 8^th^ and 9^th^ antennomeres. Careful comparison of these species with the other defining characters of the group in Jałoszyński (2018, 2019) suggest that this subgenus is, in fact a member of the North American fauna.

The subgenus Cladoconnus comprises thirty-seven extant species distributed predominantly in the Western Palaearctic region (although recent discoveries by Hoshina and colleagues (Hoshina 2004; Hoshina et al. 2018; Hoshina and Park 2020) suggest that the east Asian diversity is considerable as well) and one fossil species from ~ 40 MY Baltic amber (Jałoszyński and Perkovsky 2021). Here I report this subgenus for the first time from the western hemisphere, represented by 13 previously undescribed species. At the same time, these species require a redefinition of the subgenus, because they exhibit considerable variability in morphology, notably not all having the most conspicuous defining character of the subgenus, the carinae of the male 8^th^ and 9^th^ antennomeres.

This group also has significance from a regional, faunistic perspective, as it seems to represent a wholly undocumented high Appalachian radiation. Such endemic radiations are known in numerous other taxa, both arthropods (Barr 1979; Gusarov 2002; Miller and Wheeler 2005; Wheeler and Miller 2005) and other organisms. *Cladoconnus* are somewhat unusual among even these, however, in having no other close relatives (at least as far as currently documented) elsewhere in North America. Other regional species of *Euconnussensu stricto* (where these species might otherwise have keyed out, in e.g., O’Keefe 2001) do not appear to be closely related, most in the southeastern US exhibiting a distinct suite of sexual dimorphisms, particularly on the male abdominal ventrites. On a regional scale, these separate lineages show no geographical overlap, with *Euconnuss. str.* records coming mainly from low-lying areas in the coastal plain. Most records of the species assigned to *Cladoconnus* are from ~ 1000 m or above, and some subgroups primarily above 1500 m in the highly restricted spruce-fir habitats of the highest peaks.

In addition to describing these species, I report COI barcode sequences for most of them, several being represented by sequences from multiple localities, allowing a preliminary assessment of their internal phylogenetic relationships, as well as an even more preliminary exploration of their possible relationships among *Euconnus* species on a broader scale.

## ﻿Materials and methods

Specimens used in this paper originated in or are deposited in the following collections:

**AMNH**The American Museum of Natural History, New York;

**FMNH**The Field Museum, Chicago;

**CNCI**The Canadian National Collection of Insects, Ottawa;

**CUAC**The Clemson University Arthropod Collection, Clemson;

**UNHC** The University of New Hampshire Collection, Durham.

Most of the 679 specimens used in this study were collected as part of a larger inventory of leaf litter inhabiting arthropods. Litter samples were obtained from numerous high elevation localities (> 3300 ft or 1000 m) across five states (Virginia, North Carolina, Tennessee, South Carolina, Georgia) during the years 2015–2021, visiting most sites on two different dates, roughly in spring and fall timeframes, and collecting at least three separate litter samples on each visit. Many of the highest elevation samples come from spruce-fir forest, where litter consists of deep decomposing needles, with minor components of deciduous leaves and fine woody debris. But at lower sites, deciduous leaf litter or *Rhododendron* litters were often sampled. Litter was sifted down to the soil surface (or to a depth where litter was so decayed as to be indistinguishable from soil, where the interface was not a hard boundary), over an area of ca. 1 m^2^, through an 8-mm mesh, until a bag of ~ 6 L was filled. Samples were processed in the lab using Berlese-Tullgren funnels, running subsamples until thoroughly dry, ~ 12 hours per batch. Specimens were collected directly into 100% ethanol, and moved to -20 °C storage after each subsample was complete.

### ﻿Conventions

Despite availability of many more specimens of most species, type series are generally restricted to single localities, acknowledging the possibility of cryptic diversity within some of these species. Type specimens are all dissected males or specimens that we’ve been able to associate with dissected males through DNA sequences. Label data for primary and secondary types are quoted; data for non-type specimens are summarized. Full specimen-level data for all material examined, along with voucher codes, extraction codes, and GenBank accession numbers are detailed in an Excel supplement.

Following Hlavác and Stevanović (2013), measurements conform with the following conventions:

- **BL** – total body length is a sum of the lengths of head (anterior labral margin to summit of vertex), pronotum and elytra measured separately;
- **HW** – width of head is measured across eyes;
- **PW & PL** – pronotal width and length are the maximum widths and lengths;
- **EW** – elytral width is the maximum width;
- **
EL**– length of elytra along suture.

### ﻿Sequencing

Many specimens reported here were processed through a voucher-based high-throughput sequencing protocol in an attempt to generate a barcode database for the high Appalachian litter arthropod fauna. Subsequently, I also selected additional specimens, many dry, from older collections to complement these, representing additional localities and potential species. Most barcoded specimens were imaged prior to extraction, with images archived on a Flickr page: https://www.flickr.com/search/?user_id=183480085%40N02&text=Cladoconnus. The supplementary specimens were not imaged, but other sequencing procedures were similar. Dry specimens were removed from points by soaking in 100% ethanol. Every specimen was subdivided or punctured to permit tissue digestion, and placed in a separate well in a 96-well plate. Tissues were digested with lysis buffer and proteinase K (Omega BioTek, Norcross, GA). The liquid fraction was removed to a new plate, leaving behind the voucher remains for further dissection and archiving. Following digestion, remains of extracted specimens were recombined with any non-extracted body parts, labelled, assigned unique CUAC (Clemson University Arthropod Collection) identifiers, and curated into the CUAC. The digested tissue mixture was purified using Omega BioTek’s MagBind HDQ Blood and Tissue kit on a Hamilton Microlab Star automated liquid handling system, eluting with 150 μL elution buffer.

Analyses reported here include sequences from two separate sequencing approaches, both based on a ‘mini-barcode’, a 421 bp fragment of the mitochondrial COI gene amplified using the primers BF2-BR2 (GCHCCHGAYATRGCHTTYCC & TCDGGRTGNCCRAARAAYCA, respectively; Elbrecht and Leese 2017), corresponding to the downstream two-thirds of the standard barcoding region. Primer pairs for each well were tagged with a unique combination of forward and reverse 9 bp indexes to allow multiplexing, synthesized as part of the primer by Eurofins Genomics (Louisville, KY). These indexes were derived from a list provided by Meier et al. (2016). All PCRs were conducted in 12.5 μL volumes (5.6 μL water, 1.25 μL Taq buffer, 1.25 μL dNTP mix [2.5 mM each], 0.4 μL MgCl [50 mM], 1.5 μL each primer, 0.05–0.09 μL Platinum Taq polymerase, 1–2 μL DNA template, with a 95 °C initial denaturation for 5 minutes, followed by 35–45 cycles of 94 °C (30 sec), 50 °C (30 sec), 72 °C (30 sec), and a 5 minute 72 °C final extension on an Eppendorf Gradient Mastercycler.

Earlier barcodes (pre-2022) were generated on an Illumina MiSeq, later ones on an Oxford Nanopore MinION sequencer. For Illumina library preparation, PCR products were combined and purified using Omega Bio-Tek’s Mag-Bind Total Pure NGS Kit, in a ratio of 0.7:1 (enriching for fragments >300 bp). Illumina adapters and sequencing primers were ligated to PCR products using New England BioLab’s Blunt/TA Ligase Master Mix. The amplicon+adapter library was again purified using Mag-Bind Total Pure NGS, and quantified using a Qubit fluorometer. This library was sequenced on an Illumina MiSeq using a v.3 2×300 paired-end kit. Nanopore libraries were prepared using the ligation sequencing kit LSK-112 (Oxford Nanopore Technologies, Oxford, UK) and sequenced using a v10.4 flowcell.

Illumina reads were processed with bbtools software package (https://jgi.doe.gov/data-and-tools/bbtools/; v38.87, Bushnell et al. 2017) to merge paired read ends, remove PhiX reads, trim Illumina adapters, filter reads for the correct size, remove reads with quality score<30, cluster sequences by similarity allowing 5 mismatches (~1%), and generate a final matrix in FASTA format. Nanopore reads were basecalled using the ‘super-accurate’ algorithm of Guppy (v6.1.2), then demultiplexed using ONTbarcoder v0.1.9 (Srivathsan et al. 2021), with minimum coverage set at 5. Final barcode sequences were submitted to GenBank, under accession numbers OP779401–OP779518 (see Suppl. material 1 for details).

### ﻿Phylogeny

FASTA files from all sequencing methods were combined and aligned with the online version of Mafft v7 (Katoh et al. 2017) using the ‘auto’ strategy. To attempt to determine the placement of putative Appalachian *Cladoconnus* species among global *Euconnus* diversity, sequences were combined with other barcode-region sequences for *Euconnus* available on BOLD and GenBank, making a combined matrix of 389 sequences. These include 117 American putative *Cladoconnus*, two European *Cladoconnus*, 265 other *Euconnus* sequences (multiple individuals for many species, as well as many unidentified) representing other subgenera (*Neonapochus*, *Tetramelus*, *Napochus*, and *Euconnus* s. str., Old World and New), and five outgroups from the also-Glandulariini genus *Brachycepsis* Brendel. Phylogenetic reconstructions were performed using maximum likelihood (ML) with W-IQ-TREE v. 2.0 (Trifinopoulos et al. 2016), available at http://iqtree.cibiv.univie.ac.at. This program was used also to determine the best substitution model for the data. Analyses used a perturbation strength of 0.4. and an IQ-TREE stopping rule value at 200. Branch support is based on an ultrafast bootstrap analysis (Minh et al. 2013), run with 1,000 bootstrap replicates with a minimum correlation coefficient of 0.99.

## ﻿Results

### ﻿Taxonomy

**Genus *Euconnus* Thomson, 1859**

#### ﻿Subgenus Cladoconnus

Taxon classificationAnimaliaColeopteraStaphylinidae

Reitter, 1909

457818E2-6B0F-5801-BBA8-8C70B58D362E

Cladoconnus Reitter, 1909: 226, as subgenus of Euconnus Thomson. Type species: Scydmaenusmotschulskii Motschulsky, 1837 (subsequent designation by Newton and Franz 1998: 145).
Euconnus (Cladoconnus) Reitter: Hlavác and Stevanović 2013 (diagnosis); Jałoszyński 2018 (diagnosis).

##### Diagnosis.

**Figure 1.**
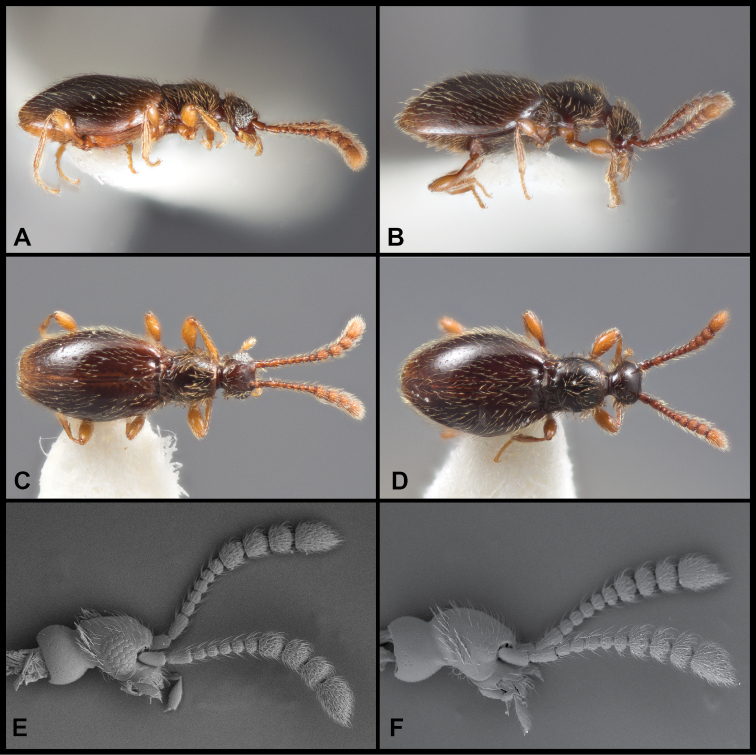
Habitus and character photos of *Euconnusmegalops***A** male, lateral view **B** female, lateral view **C** male, dorsal view **D** female, dorsal view (BgBld.B.349) **E** SEM of male head, lateral **F** SEM of female head, lateral.

Previous diagnoses of Palaearctic *Cladoconnus* have focused primarily on the presence in males of serrulate carinae on the inner margins of antennomeres VIII and IX, a character not known elsewhere in *Euconnus*. Jałoszyński (2018) further noted the unique presence of a bell-shaped, setose pronotum with 3 basal foveae in an inward-pointing triangular arrangement on each side of a short median basal pronotal carina, straddling short sublateral carinae. He also noted a unique mandibular shape in which the outer margin is interrupted where the apical portion narrows abruptly. The species described here do not entirely conform to this diagnosis, showing variability in most of these characters among them. All are small, with total body lengths ranging from 1.3 to 1.7 mm, not differing much between the sexes (Table 1). The highly distinctive male antennal carinae are evident in most of these species (Figs 4E, 6, 9E, 14C, 16C), but are completely absent in a couple that otherwise seem to be related by other morphological characters and by DNA sequences (Figs 1E, 8A, 13E). Most are consistent in having a bell-shaped pronotum, narrowed basally in some, with the triangle pattern of posterior foveae and at least short sublateral carinae (Figs 4F, 9F). A median basal pronotal carina is evident in several (e.g., Fig. 4F), but in others it is so weak as to be essentially absent (e.g., Fig. 9F). Only one species (*E.vetustus*) exhibits the distinctive bi-arcuate mandible, the others appearing to have simply arcuate mandibles. Similar to all Palaearctic species, American *Cladoconnus* have male genitalia with complex, asymmetrical endophallic armature. Metathoracic wings have not been described for European species, but illustrations and descriptions of body and metathoracic shape suggest that most described species are fully winged in both sexes. Hoshina et al. (2018) notes apparent sexual dimorphism in wingedness in three Asian species. Several of the new American species are flightless in both sexes, and some appear to be flightless in females only. Females associated by sequencing show reduced eyes relative to males, and have smaller, unmodified antennomeres, in several species having the club reduced to only three antennomeres (e.g., Fig. 9D). Due to this variability, it is likely that future revision of *Cladoconnus* will be necessary, but for now these new species seem to have clear relationships to those in the Old World, and are relatively easy to recognize among New World *Euconnus*.

**Table 1.** Measurements of E. (Cladoconnus) species, in mm. Each cell has separate averages for males / females, with total number measured in the last column.

	HL	HW	PL	PW	EL	EW	BL	n
*E.megalops*	0.4/0.4	0.3/0.3	0.4/0.4	0.3/0.4	0.9/0.9	0.6/0.6	1.7/1.7	3 / 3
*E.falcatus*	0.3/0.3	0.2/0.2	0.3/0.3	0.3/0.3	0.7/0.7	0.5/0.5	1.3/1.3	3 / 3
*E.cataloochee*	0.3/0.3	0.2/0.2	0.3/0.3	0.3/0.3	0.7/0.7	0.5/0.5	1.4/1.3	3 / 3
*E.tusquitee*	0.3	0.3	0.3	0.3	0.7	0.4	1.3	1 / 0
*E.kilmeri*	0.3	0.3	0.3	0.3	0.7	0.4	1.3	1 / 0
*E.draco*	0.3/0.3	0.2/0.2	0.3/0.3	0.3/0.3	0.7/0.7	0.5/0.5	1.3/1.3	3 /2
*E.vetustus*	0.3/0.4	0.3/0.3	0.3/0.4	0.4/0.4	0.9/0.8	0.6/0.6	1.5/1.5	3 / 3
*E.attritus*	0.3/0.3	0.3/0.3	0.4/0.4	0.3/0.3	0.8/0.8	0.5/0.5	1.5/1.5	3 / 3
*E.astrus*	0.4/0.4	0.3/0.3	0.3/0.4	0.3/0.3	0.8/0.9	0.6/0.6	1.5/1.6	3 / 3
*E.vexillus*	0.4/0.4	0.3/0.3	0.4/0.4	0.4/0.4	0.9/0.9	0.7/0.7	1.7/1.7	3 / 3
*E.adversus*	0.4/0.4	0.3/0.3	0.4/0.4	0.3/0.4	0.9/0.9	0.6/0.6	1.6/1.6	3 / 3
*E.cumberlandus*	0.4/0.4	0.3/0.3	0.4/0.4	0.4/0.4	0.9/0.9	0.6/0.6	1.7/1.7	3 / 3
*E.cultellus*	0.3/0.3	0.3/0.2	0.3/0.3	0.3/0.3	0.7/0.7	0.5/0.5	1.3/1.3	3 / 3

There is little point writing a key to these species because most can only definitely be identified by male genitalia, with a few externally similar species even sympatric in a few places. There are three main morphotypes, dark and stout (*Euconnusvexillus* and *E.vetustus*), dark with rufescent highlights, more gracile (*Euconnusmegalops*), and small and pale (flightless), with a mix of modified and non-modified male antennomeres (all remaining species). All share a generally similar form of male genitalia: the basal bulb is large and voluminous, narrowing at the shoulders to a variously tapered median lobe (sensu Stephan et al. 2021; equivalent to the dorsal apical projection of Jałoszyński 2012. The opposite side (morphologically ventral) exhibits a thin, weakly sclerotized compressor plate (sensu Stephan et al. 2021; equivalent to the ventral apical projection of Jałoszyński 2012). They all have a crescent-shaped diaphragm plate sclerite, and relatively thin, weakly curving parameres, bearing a small number of apical setae. They vary much more, however, in the sclerites of the endophallus, which are always asymmetrical, including two or more long, curving, often opposing hooks or spikes. These may bear secondary hook-like processes, or other variously acute projections. In descriptions ‘upper’ and ‘lower’ are used to refer to the diaphragm and foramen sides, respectively, and ‘left’ and ‘right’ referring to the structures as drawn (upper side up), not to true morphological position.

#### ﻿ Euconnus (Cladoconnus) megalops sp. nov.

Taxon classificationAnimaliaColeopteraStaphylinidae

4680ED87-D23D-5F53-8E99-E3DFCF10FADF

https://zoobank.org/92355F93-458D-455B-B19E-CFE0D3DFE12E

Figs 1 , 2A , 3

##### Type material.

***Holotype*** ♂, deposited in FMNH: “USA: NC: Haywood Co., 35.6721°N, 83.1760°W, Smoky Mts NP, 6150’, Big Cataloochee Mt., xi.5.2020, sifted litter, M.Caterino & F.Etzler” / “[QR code] CLEMSON-ENT CUAC000135174” / “Caterino DNA Voucher Extraction MSC6486, Morphosp. BCat.B.316”. ***Paratypes*** (33, CUAC, FMNH, CNCI, UNHC) – 3 ♀, 6 ♂: same data as type; 1 ♀, 3 ♂: “USA: NC: Haywood Co., 35.6686°N, 83.1749°W, Smoky Mts NP, 5725’, Big Cataloochee Mt., vii.14.2020, sifted litter, M.Caterino, F.Etzler”; 3 ♀: “USA: NC: Haywood Co., 35.6722°N, 83.1758°W, Smoky Mts NP, 6155’, Big Cataloochee Mt., vii.14.2020, sifted litter, M.Caterino, F.Etzler”; 2 ♀: “USA: NC: Haywood Co., 35.6414°N, 83.1958°W, SmokyMtsNP, Balsam Mt.Tr., 4752’, xi.5.2020, M.Caterino & F.Etzler, Sifted litter”; 3 ♀: “USA: NC: Haywood Co., 35.6425°N, 83.2007°W, SmokyMtsNP, Balsam Mt.Tr., 5167’, xi.5.2020, M.Caterino & F.Etzler, Sifted litter”; 9 ♀, 3 ♂: “USA: NC: Haywood Co., 35.6453°N, 83.2025°W, SmokyMtsNP, Balsam Mt.Tr., 5086’, xi.5.2020, M.Caterino & F.Etzler, Sifted litter”.

##### Other material.

(229 adults, 7 larvae) **GA**: Rabun Co., Chattahoochee NF, Rabun Cliffs, 4082 ft., 11-May-2021 (7 ♀, 3 ♂); Towns Co., Chattahoochee NF, Brasstown Bald, 4495 ft., 17-Nov-2020 (1 ♀, 1 ♂); **NC**: Buncombe Co. Co., Pisgah National Forest, Big Butt Trail, 5190 ft., 19-Mar-2016 (1 ♀, 2 ♂); Cherokee Co., Nantahala National Forest, Hickory Branch trail, 4156 ft., 26-Jul-2015 (1 ♀); Clay Co., Nantahala National Forest, Riley Knob, 4330 ft., 11-May-2020 (1 ♀); Clay Co., Nantahala National Forest, Shooting Creek Bald, 4809 ft., 11-May-2020 (1 ♀); Clay Co., Nantahala National Forest, Tusquitee Bald, 4656–5015ft, 1-Sep-2020 (4 ♀, 2 ♂); Clay Co., Nantahala National Forest, Chunky Gal Trail, 4014 ft., 1-Sep-2020 (2 ♀, 1 ♂); Graham Co., Nantahala National Forest, Teyahalee Bald, 4060–4663ft., 12-Apr-2022 (2 ♀, 3 ♂); Graham Co., Nantahala National Forest, Cherohala Skyway – Wright Ck., 4702 ft., 4-May-2020 (2 ♀, 3 ♂); Graham Co., Nantahala National Forest, Huckleberry Knob, 5491–5522 ft., 4-May-2020 & 13-Oct-2020 (10 ♀, 5 ♂); Graham Co., Nantahala National Forest, jct. Indian & Santeetlah Cks., 2770–2833 ft., 24-Jun-2015 (11 ♀, 10 ♂); Graham Co., Nantahala National Forest, Joyce Kilmer Forest, 2696–2942 ft., 20-Jul-2015 (2 ♀, 5 ♂); Haywood Co., Blue Ridge Parkway National Park, Mt. Hardy, 6110 ft., 8-Sep-2020 (9 ♀, 6 ♂); Haywood Co., Pisgah National Forest, Mountains to Sea Trail, 5540 ft., 8-Sep-2020 (1 ♀); Haywood Co., Pisgah National Forest, Black Balsam Knob, 6072 ft., 7-May-2018 (1 ♀); Haywood Co., Blue Ridge Parkway National Park, Richland Balsam Mt., 6207 ft., 11-Sep-2019 (1 ♀); Haywood Co., Blue Ridge Parkway National Park, Pisgah Mt., 5245 ft., 10-Aug-2021 (1 ♀, 1 ♂); Jackson Co., Sumter National Forest, Ellicott Rock Wilderness, Bad Creek trail, 2397 ft., 3-Jun-2015 (1 ♀, 2 ♂); Jackson Co., Nantahala National Forest, Whiteside Mt., 4740 ft., 22-Jun-2022 (1 ♀); Jackson Co., Cashiers, Hwy 64, 3700 ft., 1-Feb-2020 & 16-Feb-2020 (6 ♀, 2 ♂); Jackson Co., Nantahala National Forest, Toxaway Mt., 4770 ft., 5-Aug-2020 (2 ♀, 1 ♂); Jackson Co., Blue Ridge Parkway National Park, along Blue Ridge Pkwy, 5572 ft., 11-Sep-2019 (2 ♀); Jackson Co., Balsam Mountain Preserve, Doubletop Mountain, 4839 ft., 17-Jun-2015 (3 ♀, 2 ♂); Jackson Co., Balsam Mountain Preserve, Sugarloaf Mountain, 4484 ft., 15-Jun-2015 (2 ♀); Jackson Co., Balsam Mountain Preserve, Boar ridge, 4040 ft., 16-Jun-2015 (4 ♀, 1 ♂); Jackson Co., Balsam Mountain Preserve, Dark ridge, 3290 ft., 20-Jun-2015 (2 ♀, 7 ♂); Jackson Co., Blue Ridge Parkway National Park, Waterrock Knob, 6281 ft., 29-May-2018 (1 ♀, 1 ♂); Macon Co., E Highlands, Hwy 64, 3880–3990 ft., 1-Mar-2020 (6 ♀, 2 ♂); Macon Co., Nantahala National Forest, Jones Gap, 4447 ft., 16-Jul-2015 (1 ♀); Macon Co., Nantahala National Forest, Jones Knob, 4237 ft., 28-Jul-2015 (1 ♀); Macon Co., nr. Wayah Bald, 5280 ft., 16-Mar-2016 (2 ♀); Macon Co., Nantahala National Forest, Copper Ridge Bald, 5144 ft., 9-Jul-2019 (1 ♀, 1 ♂); Macon Co., Nantahala National Forest, Cowee Bald, 4839–4942ft., 9-Jul-2019 (5 ♀, 5 ♂); Macon Co., Hwy. 64, nr. Dry Falls, 16-May-1986 (1 ♂); Madison Co., Pisgah National Forest, Camp Creek Bald, 4741 ft., 1-Mar-2022 (1 ♂); McDowell, Pisgah National Forest, Snooks Nose Trail, 2219 ft., 25-Aug-2015 (1 ♀, 2 ♂); Polk, Melrose Falls (lower), 1103 ft., 10-Aug-2021 (1 ♀, 3 ♂); Polk, Green River Game Lands, Lower Bradley Falls Tr., 1620 ft., 19-Mar-2018 (3 ♀, 1 ♂); Polk, Green River Game Lands, Green River Cove Tr., 1070 ft., 18-Mar-2018 (1 ♀); Polk, Green River Game Lands, 1740, 18-Mar-2018 Polk, Green River Game Lands, 1740 ft., 18-Mar-2018 (1 ♀, 1 ♂); Buncombe Co. Co., Blue Ridge Parkway National Park, Bull Gap, 3100 ft., 1-May-1990 (1 ♂); Swain Co., Great Smoky Mountains National Park, Clingmans Dome, 6264–6500 ft., 4-Jun-2018 & 14-Sep-2021 (4 ♂); Swain Co., Great Smoky Mountains National Park, Lakeshore Tr., Payne Ck., 1816 ft., 12-Apr-2022 (1 ♀, 2 ♂); Transylvania Co., Pisgah National Forest, Hwy 215, 1 mi. S. Blue Ridge Pkwy, 5122 ft., 7-May-2018 (1 ♀); Yancey Co., Pisgah National Forest, Woody Ridge Tr., 5086–5301ft., 15-Jun-2020 & 19-Oct-2021 (1 ♀, 2 ♂); **SC**: Greenville Co., Chestnut Ridge Heritage Preserve, 1090 ft., 8-Apr-2018 (1 ♀); Pickens Co., Eastatoe Creek Heritage Preserve, 1421 ft., 30-Apr-2015 (1 ♂); Pickens Co., Sassafras Mountain summit, 3347 ft., 10-Jun-2015 (1 ♂); Oconee Co., Sumter National Forest, Ellicott Rock Wilderness, 2113–2679 ft., 3-Jun-2015 & 4-May-2015 (5 ♀, 4 ♂); Oconee Co., Sumter National Forest, Riley Moore Falls, 900 ft., 3-Mar-2018 (2 ♀); Oconee Co., Coon Branch Nat. Area, 1950 ft., 28-Feb-2016 (3 ♀, 1 ♂); **TN**: Unicoi, Cherokee Co. National Forest, Big Bald, 5346–5430 ft., 5-Aug-2020 & 21-May-2021 (5 ♀); Blount Co., Great Smoky Mountains National Park, Whiteoak Sink, 1724 ft., 27-Oct-2021 (1 ♂); Sevier Co., Great Smoky Mountains National Park, Alum Cave Bluff Trail, 5196 ft., 25-Jun-2019 (3 ♀, 1 ♂); Sevier Co., Great Smoky Mountains National Park, Appalachian Trail nr. Newfound Gap, 5456 ft., 4-Jun-2018 (4 ♀, 1 ♂); Sevier Co., Great Smoky Mountains National Park, Off Hwy 441, 4575 ft., 12-Mar-2020 (2 ♀). **LARVAE: NC**: Swain Co., Great Smoky Mountains National Park, Clingmans Dome, 14-Sep-2021; Haywood Co., Great Smoky Mountains National Park, Big Cataloochee Mt., 5-Nov-2020; Haywood Co., Great Smoky Mountains National Park, Balsam Mt. Trail, 5-Nov-2020; Jackson Co., Blue Ridge Parkway National Park, Browning Knob, 22-Sep-2020; **TN**: Sevier Co., Great Smoky Mountains National Park, Alum Cave Bluff Tr., 28-Sep-2021; **GA**: Rabun Co., Chattahoochee NF, Rabun Cliffs, 25-Nov-2019.

##### Description.

**Figure 2.**
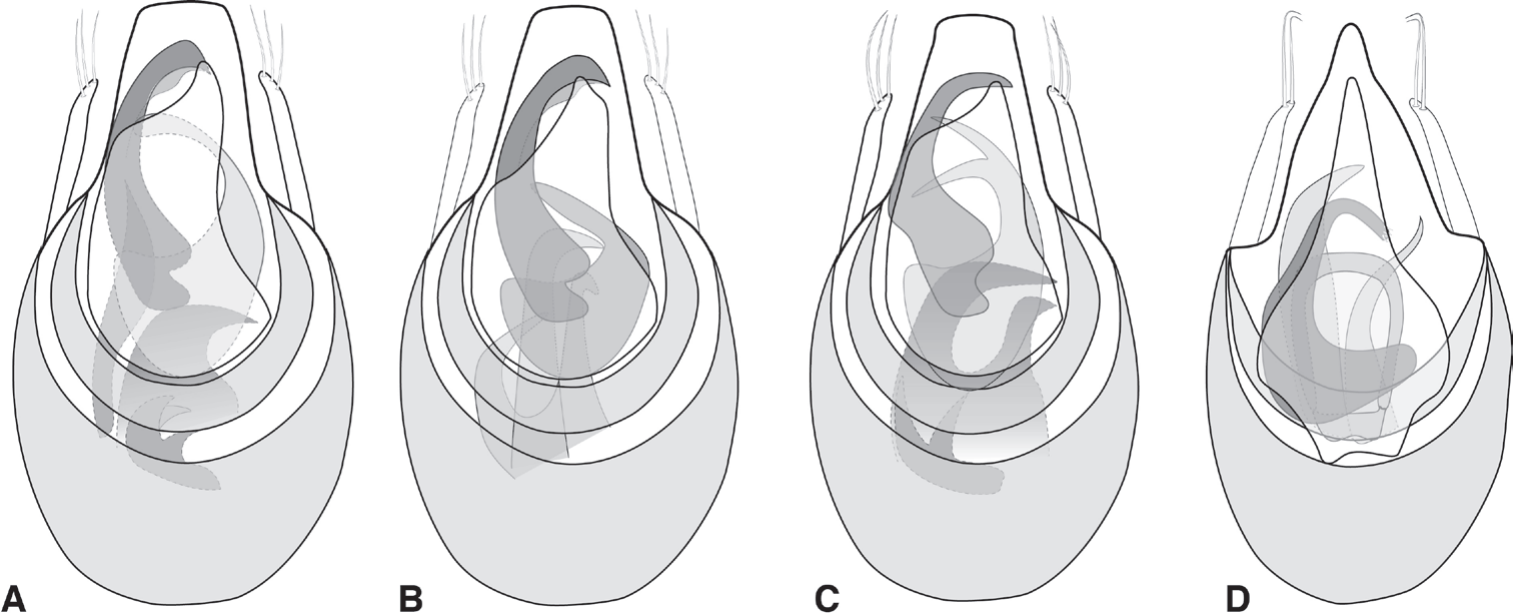
Aedeagus **A***Euconnusmegalops***B***Euconnusvexillus***C***Euconnuscumberlandus***D***Euconnusvetustus*.

Males winged, females lacking fully developed flight wings; large, elongate, generally dark but elytra reddish at humeri and often along elytral suture (Fig. 1C), female generally darker (Fig. 1D); male eyes large, protuberant, with ~ 30 large ommatidia (Fig. 1A, E); female eyes smaller, flush with side of head, comprising ~ 12 ommatidia (Fig. 1B, F); scape and pedicel similar in length, ca. as long as width of eye, antennomeres 3–6 uniformly shorter, ca. as long as wide, male antennomere VII weakly asymmetrical, slightly narrowed anterad, male antennomeres VIII-XI enlarged and elongate (longer than wide) but none carinate on anterior edges (Fig. 1E); female with antennal club tetramerous, antennomeres shorter than width (Fig. 1F). Frons and vertex with long, erect setae, each ca. as long as scape; vertex narrowed to a broadly rounded point; neck ca. one-half maximum head width; frons shallowly depressed between antennal bases; epistoma deeply depressed below antennae; labrum with anterior margin evenly rounded, with comb of short setae at middle. Pronotum densely setose at sides and anteriorly, more sparsely posterad, ‘bell-shaped’, widest just beyond middle, narrowed anterad and posterad, posterior margin slightly widening; pronotum depressed along posterior margin with very short median longitudinal carina and stronger lateral longitudinal carinae, a distinct fovea on either side between these carinae; a weak sublateral carina merging with lateral one at posterior pronotal corner, a distinct fovea between them (apparently the anterior-most of the two shown for E. (C.) motschulskii in Jałoszyński [2018]), and an additional shallow fovea below, on the side; prosternum rather short in front of procoxae, lacking median carina, with dense fringe of setae along anterior margin; elytron sparsely covered with fine setae, with pair of deep foveae at base, longitudinally weakly depressed behind these; elytral apices rounded; mesoscutellar shield hidden; mesoventrite with strong carina, setose along its crest, separating mesocoxae, extending anterad to separate apices of procoxae, posteriorly merging with metaventrite immediately behind mesocoxae; mesepimera densely setose, produced laterad mesocoxa; metacoxae narrowly separated by metaventral process; abdominal ventrites unmodified; legs generally slender, setose; protibia with only weakly expanded adhesive setae along inner margin of apical third. Aedeagus (Fig. 2A) with median lobe broadly truncate, parameres thin, rather short, straight, tapered to apex, each with three or four setae extending to or just beyond apex of median lobe; compressor plate asymmetrical, with apex obliquely truncate to weakly lobed off center; endophallus with asymmetrical armature; upper armature comprising two long curved sclerites, one longer, nearly reaching apex of median lobe at rest, abruptly curved near apex with thin inner blade bridging the apex, its inner edge concave, the other upper sclerite curving opposite, thinner, bluntly rounded at apex; lower armature comprising three separate processes: one basal, short, strongly curved, and with a bifid or trifid apex; one longer, medial, broadly hooked; the third lateral and more slender, elongate, apex acute and variously straight or bending mediad.

**Figure 3.**
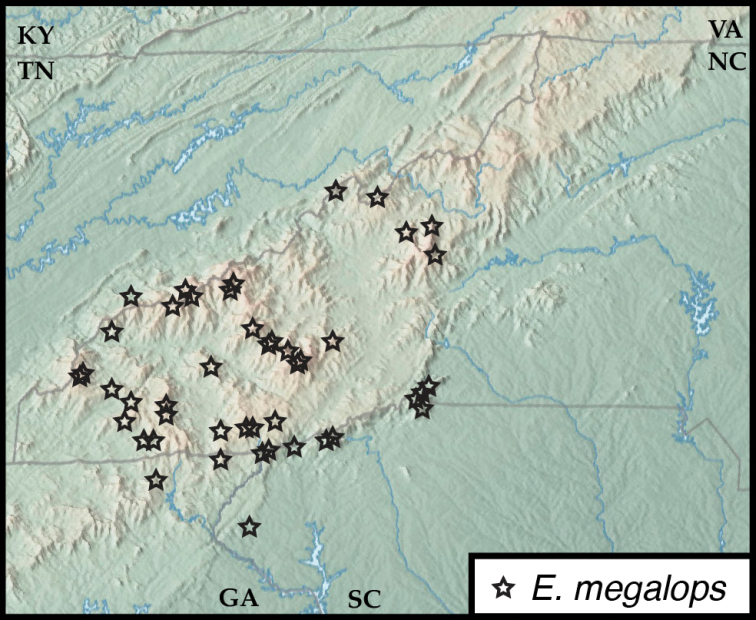
Map of collecting records for Euconnus (Cladoconnus) megalops.

##### Distribution.

This species is the most abundant and widespread of the Appalachian *Cladoconnus* species, occurring from Brasstown Bald in the southwest, northeast to Celo Knob in the Black Mts. It also exhibits the widest elevational range of the species, from ca. 900 ft in upstate South Carolina, all the way to the top of Clingmans Dome at 6500 ft.

##### Remarks.

This species’ morphology is relatively invariant across its broad range. The upper sclerites of the endophallic armature vary slightly in apical curvature and shape, but without obvious geographic trends. Similarly, none of these variants correspond to a geographically dispersed, divergent genetic subclade within the broader species, a peculiar result that merits further investigation.

This species name refers to the conspicuously enlarged eyes of the males.

#### ﻿ Euconnus vexillus sp. nov.

Taxon classificationAnimaliaColeopteraStaphylinidae

D9670F02-E3A8-5A71-953F-42B11CFD350C

https://zoobank.org/47ED180B-4423-46AC-8CA4-3BB1FEDCDB19

Figs 2B , 4 , 5

##### Type material.

***Holotype*** ♂, deposited in FMNH: “USA:SC: Greenville Co. 35.1523°N, 82.2814°W, Chestnut Ridge Heritage Preserve, vi.05.2015, S. Myers, Hardwood litter” / “[QR code] CLEMSON ENT CUAC000026944”. ***Paratypes*** (10, CUAC, FMNH) – 2 ♂, 2 ♀: same data as type; 1 ♂, 1 ♀: “USA:SC: Greenville Co. 35.1518°N, 82.2839°W, Chestnut Ridge Heritage Preserve, vi.05.2015, S. Myers, Hardwood litter”; 1 ♀: “USA:SC: Greenville Co. 35.1501°N, 82.2820°W, Chestnut Ridge Heritage Preserve, vi.05.2015, S. Myers, Hardwood litter”; 2 ♀: “USA:SC: Greenville Co. 35.1406°N, 82.2790°W, Chestnut Ridge Heritage Preserve, vi.05.2015, S. Myers, Hardwood litter”; 1 ♀: “USA:SC: Greenville Co. 35.1506°N, 82.2799°W Chestnut Ridge Heritage Preserve, iv.08.2018, M. Caterino & L. Vásquez-Vélez, sifted litter”.

##### Other material.

(26) **WV**: Mercer Co., Camp Creek State Forest, 23-Jul-1971, leaf litter (4 ♂, 11 ♀); ‘Black Mts’, x.1901 (3 ♂); **NC**: Caldwell Co., Grandfather Mt. State Park, Nuwati Trail, 4020 ft., 17-May-2021 (1 ♂); McDowell Co., Pisgah National Forest, Mackey Mountain Trail, 3433 ft., 25-Aug-2015 (2 ♂); McDowell Co., Pisgah National Forest, Snooks Nose Trail, 1998 ft., 25-Aug-2015 (3 ♀, 1 ♂); Polk Co., Green River Game Lands, 1740 ft., 18-Mar-2018 (1 ♂).

##### Diagnostic description.

**Figure 4.**
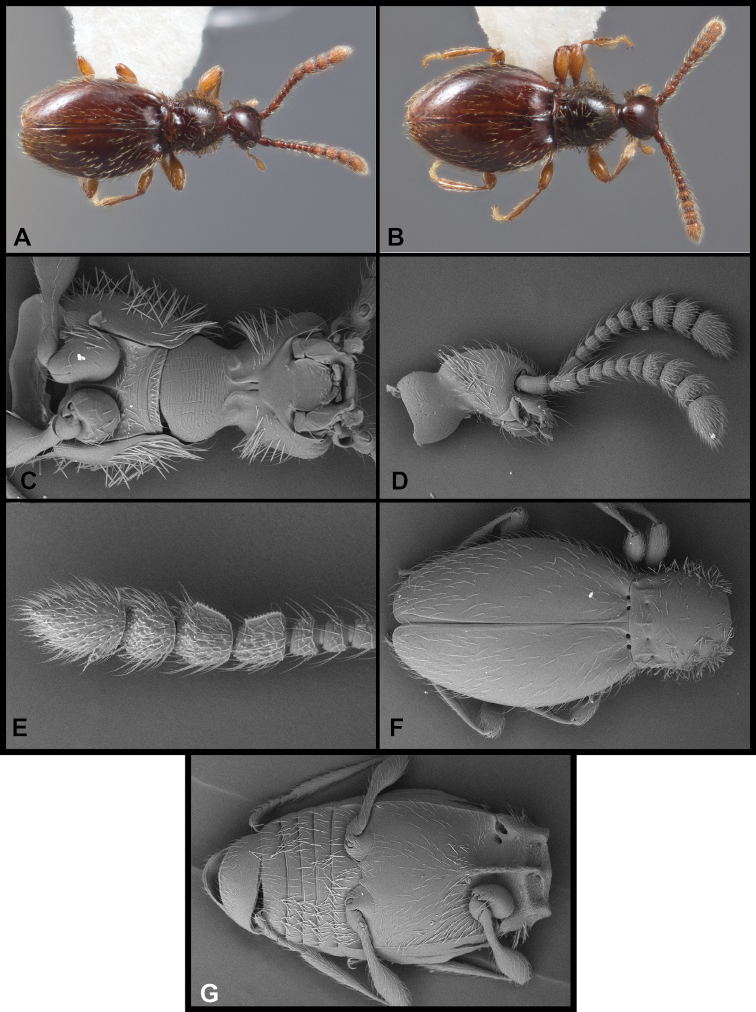
Habitus and character photos of *Euconnusvexillus***A** male, dorsal view **B** female, dorsal view **C** SEM of mouthparts, ventral view **D** SEM of female head, lateral **E** SEM of male antenna, lateral **F** SEM of elytra and pronotum **G** SEM of meta- and mesoventrites.

This species is very similar to the preceding species and can best be distinguished by male genitalic and antennal characters. Like the preceding, males are winged, while females appear not to be. A few noteworthy external differences can also be cited: males with distinct carinae on antennomeres VIII and IX (Fig. 4E); female antennae less distinctly tetramerous, with antennomere VIII intermediate in size between antennomeres VII and IX (Fig. 4D); body darker, stout, with only faintly rufescent elytral humeri, most distinctly lighter along posterior half of elytral suture; male eyes smaller, less protuberant, with only ~ 25 ommatidia; median basal carina of pronotum slightly better developed to base (Fig. 4F); aedeagus (Fig. 2B) with median lobe broadly truncate at apex; parameres short, tapered, bearing three apical setae; compressor plate strongly asymmetrical, rather short; endophallic armature with strong pair of upper processes: the left long and strongly hooked apically, with a thin inner laminar blade, its inner edge concave; the right shorter, more strongly curved toward middle of longer process; lower endophallic armature consisting of three hook-like processes of varying lengths, the lateral-most rather short and strongly curved, the medial-most of intermediate length, the one between them the longest, its tip just visible between bases of upper processes (in ‘dorsal’ view).

**Figure 5.**
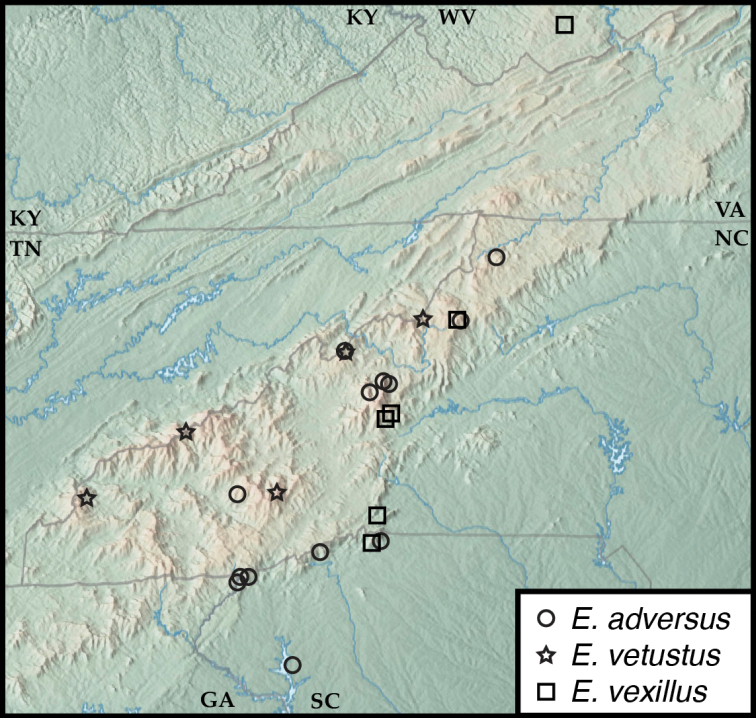
Map of collecting records for Euconnus (Cladoconnus) vexillus (squares), E. (C.) vetustus (stars), and E. (C.) adversus (circles).

##### Distribution.

This species has been found at several widely scattered localities east and northeast of the Asheville Depression, from the headwaters of the French Broad near the southern end of its range, at Chestnut Ridge, South Carolina, to southern West Virginia in the northeast. Most of its occurrences are at middle elevations, from 1090 to 4020 ft, most below 2000.

##### Remarks.

This species is similar and, judging by male genitalia, closely related to *E.megalops*. Both have similar opposing hooked upper endophallic sclerites, though the shorter right one (Fig. 2B) of *E.vexillus* is distinctive. Moreover, the lower trio of endophallic sclerites are quite distinct in *E.vexillus*, with three hooks projecting distad to varying lengths. In males, the carinae of antennomeres VII and IX will immediately distinguish this species from *E.megalops*, and the only weakly tetramerous club of females can be used to distinguish most of those, although they are similar to darker females of *E.adversus*.

This species name means ‘standard (or flag)-bearer’, referring to its possession of *Cladoconnus*-typical carinae, borne proudly on its antennae.

#### ﻿ Euconnus cumberlandus sp. nov.

Taxon classificationAnimaliaColeopteraStaphylinidae

C07CBB04-7ACC-57E7-8381-FA4224B24578

https://zoobank.org/8E94B9B6-C01A-4460-AC19-4E34FFFE452D

Figs 2C , 6 , 7

##### Type material.

***Holotype*** ♂, deposited in FMNH: “Fall Creek Falls S.Pk., Van Buren Co., TENN, 13.X.1962” / “*Rhododendron* duff, H.R.Steeves, leg.” / “Caterino DNA Voucher Extraction MSC12284”. ***Paratypes*** (9, FMNH, CUAC, CMNC): 2 ♂, 2 ♀: same data as type; 3 ♀, 2 ♂: “Fall Creek Falls S.Pk., Fall Creek Falls Pit, Bledsoe Co., TENN., 14.X.1961” / “*Rhododendron* duff, H.R.Steeves, leg.”.

##### Other material.

(12, CMNC, CUAC, FMNH) **TN**: Grundy Co., Savage Gulf State Natural Area, 1150ft., 4-May-2021, Litter – bottom of canyon (2 ♀); Pickett Co., Jamestown, Jordan Motel, 16-Jun-1962, forest floor near falls (1 ♀); **GA**: Dade Co., Cloudland Canyon State Park, 16-May-1972, rhododendron litter (2 ♂, 5 ♀); Dade Co., Cloudland Canyon State Park, 7-Aug-1962, forest floor (1 ♂, 1 ♀).

##### Diagnostic description.

**Figure 6.**
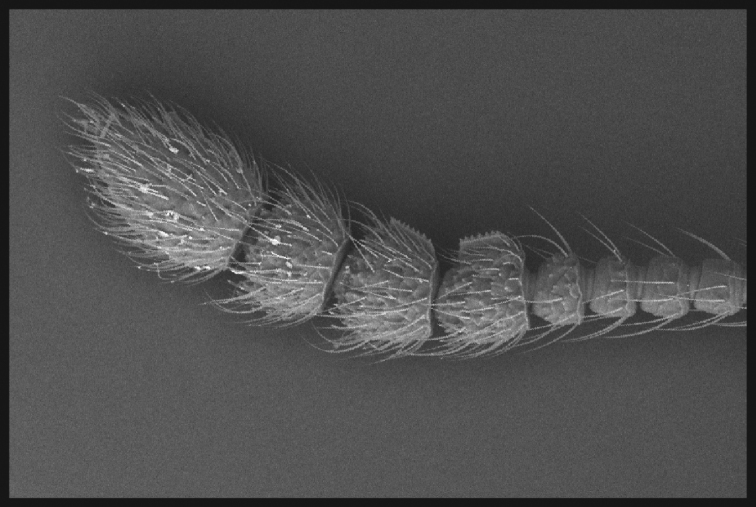
Antennal carina of *E.cumberlandus*.

This species is very similar to the preceding species and can best be distinguished by male genitalic characters. Like the preceding, males are winged, while females appear not to be. The antennal carinae of male antennomeres VIII and IX are present but rather weakly developed (Fig. 6), with neither quite spanning the entire length of the antennomere itself; female antennae less distinctly tetramerous, with antennomere VIII intermediate in size between antennomeres VII and IX; body darker, stout, with only faintly rufescent elytral humeri, most distinctly lighter along posterior half of elytral suture; male eyes smaller, less protuberant, with only ~ 25 ommatidia; aedeagus (Fig. 2C) with median lobe broad, apex weakly rounded, slightly narrowed subapically; parameres short, tapered, bearing three apical setae; compressor plate asymmetrical, produced to subacute tip on right side; endophallic armature with strong pair of upper processes: the left long and strongly hooked apically; the right shorter, strongly bifid, its apices extending beneath left process; lower endophallic armature consisting of four hook-like processes of varying lengths, two longer and moderately to strongly hooked, two shorter ones borne on a single sclerite, apices directed distad.

**Figure 7.**
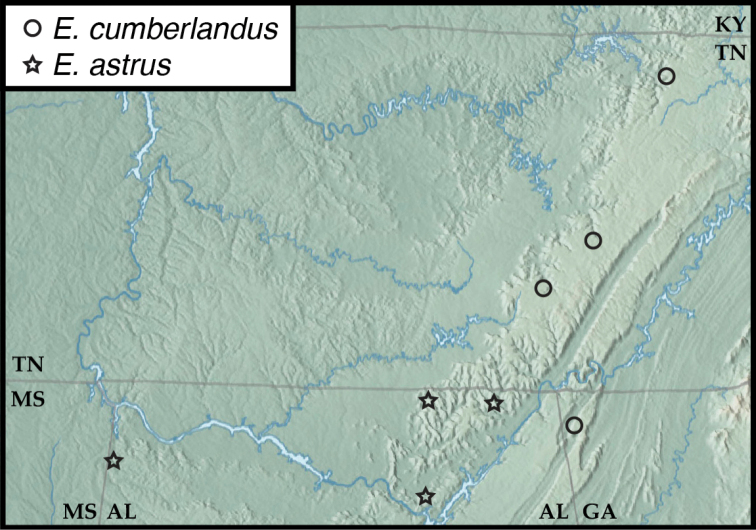
Map of collecting records for Euconnus (Cladoconnus) cumberlandus (circles) and E. (C.) astrus (stars).

##### Distribution.

This species is known from northwestern Georgia to north-central Tennessee. Two of the Tennessee localities, including the northernmost, are represented only by females, and they can be assigned only tentatively to this species.

##### Remarks.

This species is similar to and apparently closely related to *E.megalops* and *E.vexillus*. The only sequenced specimen of this species is a female from Savage Gulf, TN, a locality not yet represented by males. Sequences from other localities, including the type locality would help confirm the species’ unity as circumscribed here.

#### ﻿ Euconnus vetustus sp. nov.

Taxon classificationAnimaliaColeopteraStaphylinidae

61EC56A5-1D78-5C29-9A4D-5F2ECF2B1677

https://zoobank.org/417B9BE8-BE69-4792-926B-3B4FA19CD3DD

Figs 2D , 5 , 8

##### Type material.

***Holotype*** ♂, deposited in FMNH: “USA: NC: Mitchell Co., 36.1038°N, 82.0809°W, PisgahNF, vi.8.2020 Grassy Ridge Bald, 6083’, M. Caterino, deciduous shrub litter” / “[QR code] CLEMSON-ENT CUAC000004025” / “Caterino DNA Voucher Extraction MSC4491, Morphosp. GrB.A.336”. ***Paratypes* (4)** – 2 ♀, 2 ♂: same data as type.

##### Other material.

(7) – **NC**: Graham Co., Nantahala National Forest, Huckleberry Knob, 5511 ft., 13-Oct-2020 (1 ♂); Haywood Co., Pisgah National Forest, Black Balsam Knob, 6072 ft., 7-May-2018 (1 ♂); Yancey Co., Pisgah National Forest, Devils Gap, 3813 ft., 24-Aug-2015 (2 ♂); ‘Black Mts’ (1 ♀); **TN**: Unicoi Co., Cherokee National Forest, Big Bald, 5430–5464 ft., 5-Aug-2020 & 21-May-2021 (1 ♀, 2 ♂); Sevier Co., Great Smoky Mountains National Park, Off Hwy 441, 4575 ft., 12-Mar-2020 (1 ♀).

##### Diagnostic description.

**Figure 8.**
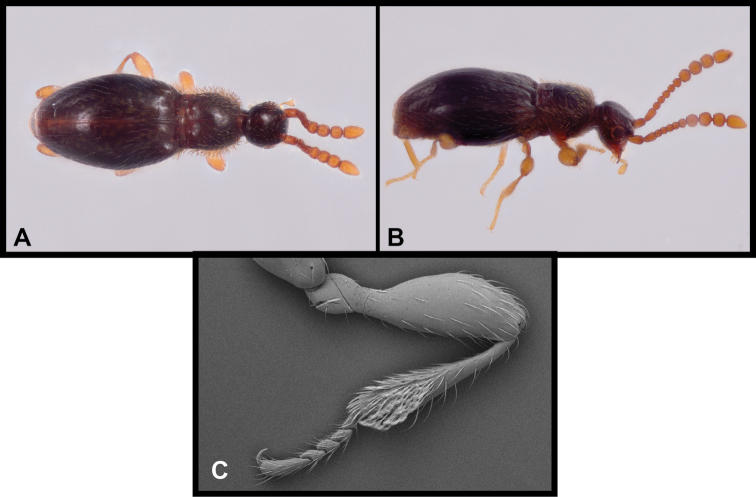
Habitus and character photos of *Euconnusvetustus***A** male habitus, dorsal (BBK.A.020) **B** male habitus, lateral (GRB.A.336) **C** SEM of protibial setae.

This species is similar to the preceding species being dark in color and wing-dimorphic, and can best be distinguished by male genitalic characters. External differences, however, include smaller body size, entirely dark coloration (Fig. 8A); head, especially crest of vertex, rounder; frons only weakly depressed between antennal bases; male and female eyes do not differ appreciably in size, both having ~ 20 ommatidia; the protibia of both sexes is shorter, widened apically, and bears conspicuously modified setae on the apical half (Fig. 8C); male antennal club tetramerous, with the club segments slightly longer than wide (Fig. 8A), no antennomeres bearing carinae; female antenna shorter, with club 4-segmented, club segments ca. as long as wide (Fig. 8B); aedeagus (Fig. 2D) with median lobe narrowed to narrowly rounded apex; rather straight parameres bear two apical setae extending just to apex of median lobe, these setae finely bent inward at apex; compressor plate symmetrical, narrow, reaching nearly to apex of median lobe; upper endophallic armature with one short curved hook, not extending beyond shoulders of tegmen, its apex blunt and distinctly fringed (at higher magnification); immediately internal to it is a rather broadly rounded thin plate (the apical margin of which is strongly sclerotized, appearing as if maybe a second opposing hook); lower endophallic armature with a long bifid process extending from deep in the basal bulb to beyond its shoulders, the inner blade thin, curving weakly upward, the outer blade longer, reaching to near apex of compressor plate, its apex finely fimbriate.

##### Distribution.

This species occurs over a relatively broad but disparate range, spanning the Asheville Depression, from the Unicois (Huckleberry Knob) in the southwest to the Roan Highlands and Big Bald in the northeast. So far it has not been found in the Black Mts or on Grandfather Mountain. Known occurrences are all at higher elevations, from 4575 to 6100 ft.

##### Remarks.

Individuals from across the broad range of this species show considerable genetic variation, and the male genitalia do show slight variation. Northern examples (Big Bald, Roan Highlands) exhibit a more strongly hooked upper endophallic process. A few specimens, from scattered localities (e.g., Smokies, Balsam Mts., and Roan Highlands), have the bi-arcuate mandibles described for European *Cladoconnus*. But this character varies as well; specimens from Big Bald, NC, have mandibles that are less distinctly arcuate, as well as longer and more slender. Male genitalia have been reexamined with this in mind, but no corresponding differences emerge. Further genetic work and longer series of males may justify separating some of these. Females from Devil’s Gap, NC are light in color (perhaps teneral) but match in other characters, including DNA.

The name of this species comes from the Latin for ‘old’, referring to the possibility the species has inhabited the area for a long time, as judging by its broad distribution, only distant relation to the rest of the species described here, and deep genetic divergences among populations.

#### ﻿ Euconnus falcatus sp. nov.

Taxon classificationAnimaliaColeopteraStaphylinidae

C4BD2E93-F75A-595D-9556-70DDDE36384F

https://zoobank.org/868CC7AB-B90A-4337-AE0E-F8329719CAAC

**Figure 9.**
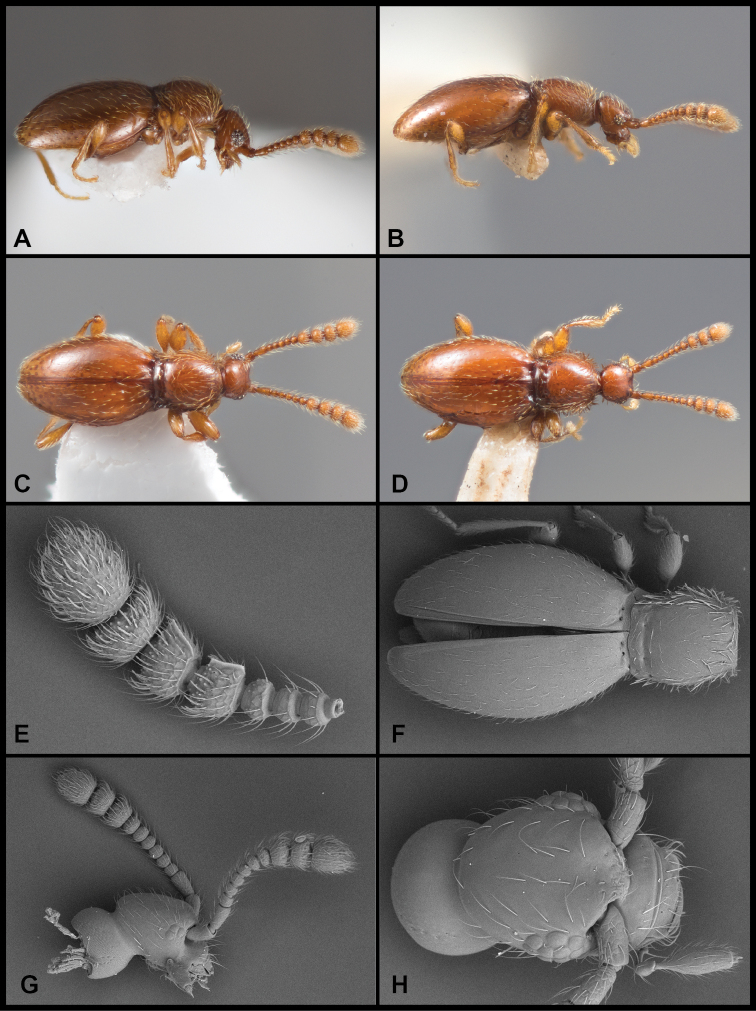
Habitus and character photos of *Euconnusfalcatus***A** male, lateral view **B** female, lateral view **C** male, dorsal view **D** female, dorsal view **E** SEM, male antenna **F** SEM, pronotum and elytra **G** SEM, female head, lateral view **H** SEM, male head, anterior view.

**Figure 10.**
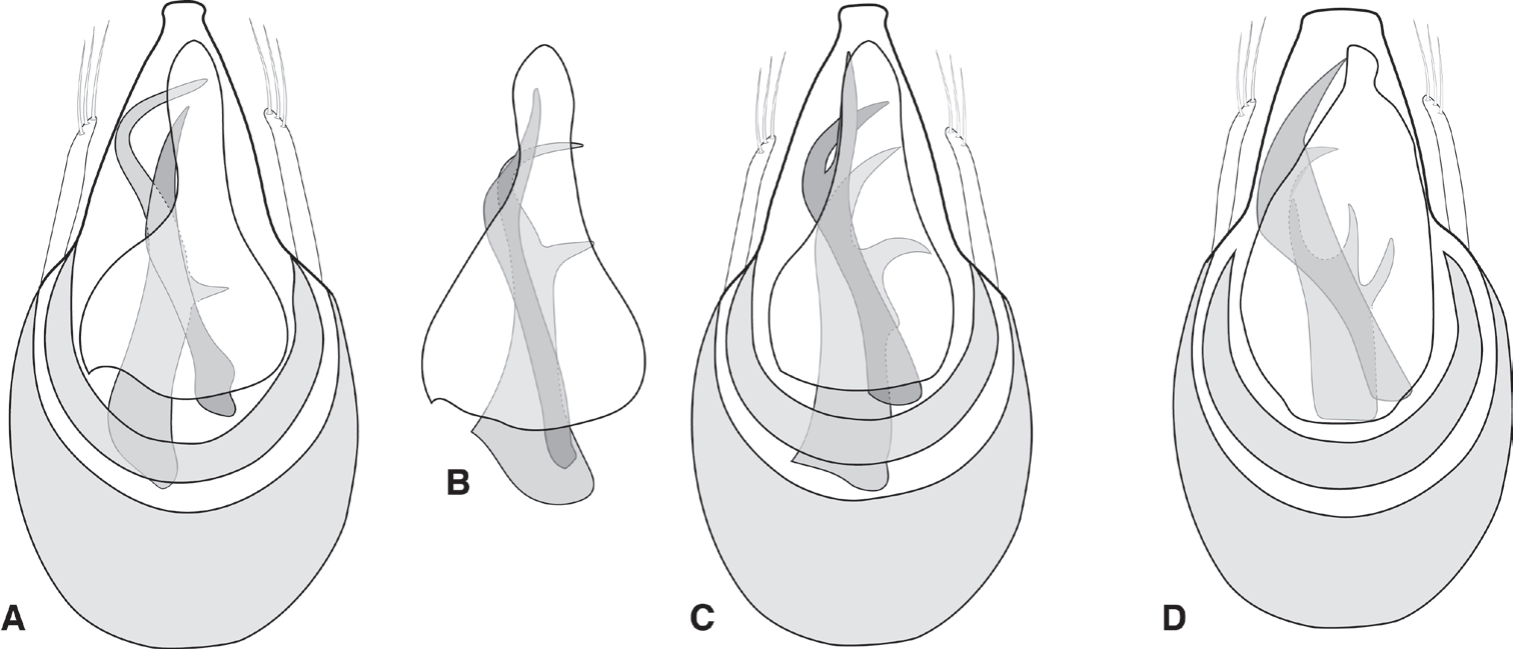
Aedeagus. **A***Euconnusfalcatus***B** compressor plate and upper endophallic armature of *Euconnusfalcatus* (Plott Balsams variant) **C***Euconnuscataloochee***D***Euconnuskilmeri*.

**Figure 11.**
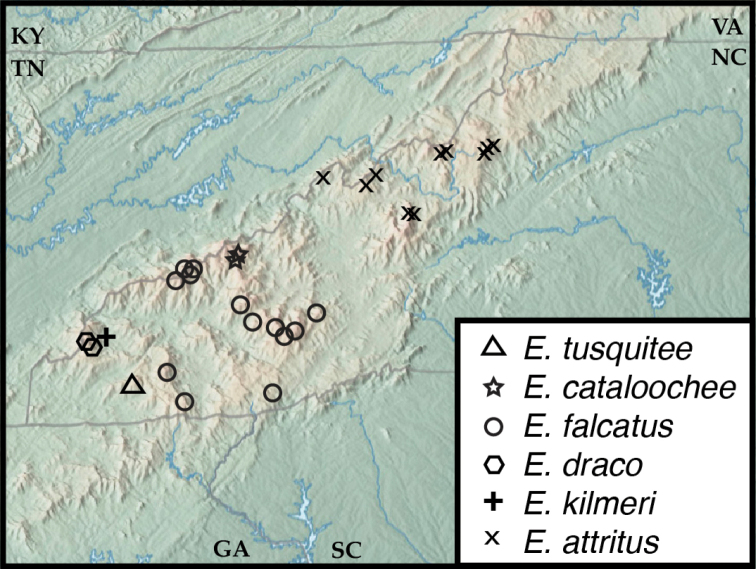
Map of collecting records for Euconnus (Cladoconnus) falcatus complex. E. (C.) falcatus (circles), E. (C.) cataloochee (stars), E. (C.) tusquitee (triangules), E. (C.) draco (hexagons), E. (C.) kilmeri (plus sign), and E. (C.) attritus (x).

Figs 9 , 10A, B , 11

##### Type material.

***Holotype*** ♂, deposited in FMNH: “USA: NC: Swain Co., 35.5566°N, 83.4966°W, SmokyMts NP, 6272’, Clingmans Dome, sifted litter, ix.14.2021, M.Caterino & E.Recuero” / “[QR code] CLEMSON-ENT CUAC000156725” / “Caterino DNA Voucher Extraction MSC7899, Morphosp. CD.B.307”. ***Paratypes*** (40, CUAC, CNCI, FMNH, UNHC) – 1 ♂: “USA: NC: Swain Co., 35.5613°N, 83.5006°W, SmokyMts NP, 6364’, ClingmansDome, W. slope, sifted litter, vi.5.2018, M.Caterino” / “[QR code] CLEMSON-ENT CUAC000079152” / “Caterino DNA Voucher Extraction MSC2834 Morphosp. CD.A.021”; 1 ♀, 3 ♂: “USA: NC: Swain Co., 35.5824°N, 83.3979°W, SmokyMtsNP,offHwy 441, 4610’, iii.12.2020, M.Caterino & F.Etzler, sifted litter”; 1 ♀: “USA: TN: Swain Co., 35.6237°N, 83.4163°W, SmokyMtsNP,offHwy 441, 4575’, iii.12.2020, M.Caterino & F.Etzler, sifted litter” / “[QR code] CLEMSON-ENT CUAC000110837” / “Caterino DNA Voucher Extraction MSC4193 Morphosp. Hwy.A.006”; 1 ♀, 8 ♂: “USA: TN: Sevier Co., 35.6160°N, 83.4149°W, SmokyMts NP, 5456’, App.Tr. E of Newfound Gap, sifted litter, vi.5.2018, M.Caterino”; 2 ♀, 9 ♂: “USA: TN: Sevier Co., 35.6308°N, 83.3904°W, SmokyMts NP, 6190’, Mt. Kephart, vi.5.2018, M.Caterino”; 1 ♂: “USA: TN: Sevier Co., 35.6311°N, 83.3895°W, SmokyMts NP, 6183’, Mt. Kephart,sifted litter, vi.5.2018, M.Caterino” / “[QR code] CLEMSON-ENT CUAC000079110” / “Caterino DNA Voucher Extraction MSC2790 Morphosp. MK.A.009”; 1 ♂: “USA: TN: Sevier Co., 35.6311°N, 83.3893°W, SmokyMts NP, 6187’, Mt. Kephart, sifted litter, ix.14.2021, M.Caterino & E.Recuero” / “[QR code] CLEMSON-ENT CUAC000156767” / “Caterino DNA Voucher Extraction MSC7941 Morphosp. MK.B.482”; 4 ♀, 4 ♂: “USA: TN: Sevier Co., 35.6425°N, 83.4427°W, SmokyMts NP, 5196’, Alum Cave Tr., vi.25.2019, M.Caterino & M.Ferro, sifted mixed litter”; 1 ♂: “USA: TN: Sevier Co., 35.6529°N, 83.4378°W, SmokyMts NP, 6467’, Mt LeConte, vi.25.2019 M.Caterino & M.Ferro, sifted conifer litter” / “[QR code] CLEMSON-ENT CUAC000079302” / “Caterino DNA Voucher Extraction MSC3483 Morphosp. MLc.009”; 1 ♀, 1 ♂: “USA: TN: Sevier Co., 35.6541°N, 83.4364°W, SmokyMts NP, 6588’, Mt LeConte, ix.28.2021, M.Caterino, sifted litter”; 1 ♂: “NC Gr.Sm.Mts.N.P., Clingmans Dome, 1950–2020m 2.VI.86 A.Smetana” (CMNC); 1 ♂: “TENN:Great Smoky Mts.St.Pk.: Newfound Gap” / “X.24.1969 W.Shear + F.Coyle leg.” / “FM(HD) 69–62 Spruce litter” (FMNH).

##### Other material.

(36, CUAC, UNHC, CNCI) – **NC**: Haywood Co., Pisgah National Forest, Black Balsam Knob, 6033–6072ft, 7-May-2018 & 20-Oct-2020 (3 ♂); Haywood Co., Pisgah National Forest, Mountains to Sea Trail, 5540ft., 8-Sep-2020 (1 ♂); Haywood Co., Blue Ridge Parkway National Park, Mt. Pisgah, 5420ft., 13-Sep-2022 (1 ♀, 1 ♂); Jackson Co., Blue Ridge Parkway National Park, Mt. Lyn Lowry, 6097 ft., 15-Jun-2021, sifted litter (1 ♀); Jackson Co., Blue Ridge Parkway National Park, Mt. Lyn Lowry, 6205 ft., 22-Sep-2020, sifted litter (1 ♀); Jackson Co., Blue Ridge Parkway, National Park, along Blue Ridge Pkwy, 5572ft., 11-Sep-2019 (1 ♀, 1 ♂); North Carolina, Jackson Co., Blue Ridge Parkway National Park, Browning Knob, 6140–6221, 22-Sep-2020 & 29-May-2018 (3 ♀, 1 ♂); Jackson Co., Blue Ridge Parkway National Park, Waterrock Knob, 6059, 29-May-2018 (4 ♀, 4 ♂); Jackson Co., Blue Ridge Parkway National Park, Waterrock Knob, 6281, 29-May-2018; Jackson Co., Balsam Mountain Preserve, Doubletop Mountain, 5396–5480, 7-Feb-2015 & 15-Jun-2015 (4 ♂); Jackson Co., Nantahala National Forest, Toxaway Mt., 4750, 5-Aug-2020 (1 ♂); Jackson Co., Wolf Mt. overlook, 26-May-1986 (3 ♂); Jackson Co., Blue Ridge Parkway National Park, 5572, 11-Sep-2019 (1 ♀); Macon Co., Nantahala National Forest, Copper Ridge Bald, 5144, 9-Jul-2019 (1 ♂); Macon Co., Hwy. 64, nr. Dry Falls, 16-May-1986 (2 ♀, 1 ♂); Macon Co., Coweeta Hydrological Lab, Shope Fork, 3200, 28-May-1983 (1 ♂).

##### Description.

Body rufescent (Fig. 9A-D), weakly translucent; head and elytra sparsely setose, pronotum densely setose, especially at sides; head with frons weakly depressed between antennal bases (Fig. 9H), vertex convex, neck just over one-half maximum head width; eyes of male moderately large, protuberant, comprising ca. 15 distinct ommatidia (Fig. 9H), situated immediately behind antennal insertions, width slightly more than that of tempora behind; eyes of female smaller (Fig. 9G), less prominent, comprising < 8 ommatidia, width only one-third that of tempora; antennae inserted under blunt frontal shelf; antennae of male with scape and pedicel similar in length, ca. twice length of antennomeres III-VI, antennomere VII slightly larger, asymmetrical, slightly produced on anterior margin, antennomeres VIII-XI distinctly larger, antennomeres VIII and IX with longitudinal carinae on anterior margin (Fig. 9E), that of VIII more or less parallel to antennal axis, that of IX more strongly produced at apex, antennomeres VIII-X similar in length, terminal antennomere ca. twice as long; female antennae shorter, club composed of only three short, wide antennomeres (Fig. 9D, G); pronotum weakly bell-shaped, with sides arcuate (Fig. 9F), widest ca. one-fourth behind straight anterior margin, narrowed posteriorly, similar in width to anterior, with short longitudinal carinae on either side, transversely depressed between, with small submedian foveae on either side of low median ridge (median basal carina absent), and two small foveae outside lateral carina, pronotal sides very densely bristled; mesoscutellar shield hidden; elytra broadly rounded, widest just anterad middle, each with two small basal foveae, sparse setae randomly arranged; flight wings poorly developed or absent in both sexes; abdominal ventrites without secondary sexual differences; aedeagus (Fig. 10A) broadly rounded basally, apex of median lobe narrowed, pinched before apex, subtruncate; parameres thin, close to median lobe, with two long apical setae extending beyond median lobe apex; dorsal diaphragm crescent-shaped; compressor plate thin, narrowly rounded at apex, slightly asymmetrical; endophallus with two asymmetrical primary sclerites, an upper one almost linear, weakly sinuous to acute apex, the lower one strongly sickle-shaped, bearing a single conspicuous and sometimes an additional small secondary processes on its inner surface.

##### Distribution.

*Euconnusfalcatus* exhibits a wide distribution, especially considering its flightlessness, including the Great Smoky Mountains, continuing along the high Blue Ridge Parkway corridor into the Plott Balsams and Great Balsams, as well as slightly lower parts of the Nantahala and Cowee Mountains. In elevational range it occurs from 3200 ft at Coweeta to its highest occurrences on Clingmans Dome and Mt. LeConte above 6500 ft.

##### Remarks.

A more complete description of this species is provided to serve as a general description for several subsequent species that differ in few or no obvious external characters, including *E.cataloochee*, *E.kilmeri*, *E.draco*, *E.tusquitee*, *E.attritus*, *E.cultellus*, *E.adversus*, and *E.astrus*. The modified male antennomeres differ significantly in a couple of these (*E.adversus* and *E.astrus*), but only slightly or not at all in the others. *Euconnusfalcatus* can only be unambiguously distinguished from the others by male genitalic characters. Specifically, the lower endophallic sclerite is long, sickle-shaped, and bears a small secondary hook near its midpoint. The apex of the median lobe is more narrowly knobbed than most others, and the apex of the compressor plate is only subtly asymmetrical. There appear to be some differences in exact shapes of endophallic sclerites among localities (e.g., Fig. 10B). Some of this variation may be real, but some also appears to result from varying degree of extroversion of the lower hooked sclerite relative to the upper one.

This species name refers to the distinctively ‘sickle-shaped’ hook of the endophallic armature.

#### ﻿ Euconnus cataloochee sp. nov.

Taxon classificationAnimaliaColeopteraStaphylinidae

BC8AD954-DB2A-5E38-82F8-6778B1505E6D

https://zoobank.org/FD84FEAD-72A4-4FBD-B0D6-B3A76AA6B697

Figs 10C , 11

##### Type material.

***Holotype*** ♂, deposited in FMNH: “USA: NC: Haywood Co., 35.6721°N, 83.1760°W, Smoky Mts NP, 6150’, Big Cataloochee Mt., xi.5.2020, sifted litter, M.Caterino & F.Etzler” / “[QR code] CLEMSON-ENT CUAC000135173” / “Caterino DNA Voucher Extraction MSC6485 Morphosp. BCat.B.315”. ***Paratypes*** (32 – CUAC, FMNH, CNCI, UNHC): 7 ♀, 7 ♂: same data as type; 5 ♀, 5 ♂: “USA: NC: Haywood Co., 35.6453°N, 83.2025°W, SmokyMtsNP, Balsam Mt.Tr., 5086’, xi.5.2020, M.Caterino & F.Etzler, Sifted litter”; 1 ♀, 1 ♂: USA: NC: Haywood Co., 35.6686°N, 83.1749°W, Smoky Mts NP, 5725’, Big Cataloochee Mt., vii.14.2020, sifted litter, M.Caterino, F.Etzler”; 1 ♂: “USA: NC: Haywood Co., 35.6724°N, 83.1761°W, Smoky Mts NP, 6130’, Big Cataloochee Mt., vii.14.2020, sifted litter, M.Caterino, F.Etzler”; 3 ♀, 2 ♂: “USA: NC: Haywood Co., 35.6675°N, 83.1805°W, Smoky Mts NP, 5586’, MtSterlingTr@Lost BottomCk, vii.14.2020 M.Caterino& F.Etzler, sifted litter”.

##### Diagnostic description.

This species exhibits no obvious external differences from the preceding species and can only be distinguished by male genitalic characters. Antennomere VIII of male slightly more strongly produced at inner basal corner, slightly oblique to antennal axis; aedeagus (Fig. 10C) with median lobe slightly narrowed, pinched near apex, bluntly rounded; parameres curving slightly inward at tips, each with three apical setae; compressor plate weakly asymmetrical and narrowly rounded; endophallus with two asymmetrical primary sclerites, one long, sinuate, apically bifid, with lateral hook curving beneath a median apically directed spear; lower endophallic sclerite originating nearer base, broad at base, subdivided into two widely spaced apical hooks, curving the same direction as inner hook of upper sclerite.

##### Distribution.

This is a very restricted species, only known from a couple localities on or very near Big Cataloochee Mountain in the Smokies, and is only known above 5100 ft.

##### Remarks.

The bifid apex of the upper endophallic sclerite is similar to that of *E.cultellus*, but the subapical (lower) tip is more strongly developed, almost appearing as a separate sclerite. The two well-developed hooks on a shorter overall lower endophallic sclerite distinguish it from *E.falcatus*. Sequence differences from the latter are minimal to non-existent, suggesting either very recent ancestry or introgression, as they do seem to be sympatric in the central Great Smoky Mountains, the only locality known for *E.cataloochee*.

This species is named for its type locality, Big Cataloochee Mt., as it is known from only a small area near its summit. The name apparently comes from a Cherokee word referring to the prominent wooded ridges in this region of the Great Smoky Mountains.

#### ﻿ Euconnus kilmeri sp. nov.

Taxon classificationAnimaliaColeopteraStaphylinidae

A2F744EA-B2AD-5E80-B83C-0972F530E3F6

https://zoobank.org/435F225A-A9C8-4F63-AEE1-262A9852E969

Figs 10D , 11

##### Type material.

***Holotype*** ♂, deposited in FMNH: “USA:NC: Graham Co., 35.3433°N, 83.96207°W, Joyce Kilmer, VII.20.2015 S. Myers, Sifted litter” / “[QR code] CLEMSON-ENT CUAC000011399” / “Caterino DNA Voucher Extraction MSC11827”; ***Paratypes*** (0).

##### Diagnostic description.

This species exhibits no obvious external differences from the preceding two species, and can best be distinguished by male genitalic characters; antennal carinae slightly weaker than preceding; aedeagus (Fig. 10D) with median lobe rather short, narrowed to bluntly rounded apex, only very weakly knobbed; parameres with apices obliquely truncate, bearing three long, curved setae; compressor plate strongly asymmetrical, apex narrowly knobbed and displaced to one side; endophallus with dominant upper sclerite sickle-shaped, with long straight inner edge, outer edge strongly curved, broadest just beyond middle, bearing a secondary inner tooth directed mediad and inward; lower endophallic sclerite shorter, bearing two widely separated distally pointing spikes, and an obliquely directed subapical spike on one side.

##### Distribution.

*Euconnuskilmeri* is only known from a single locality within the Joyce Kilmer Memorial Forest in far western North Carolina. This site sits at an elevation of 2800ft.

##### Remarks.

The aedeagus of *E.kilmeri* is quite distinct in the shape of the strongly asymmetrical compressor plate, the long, straight inner edge of the upper endophallic sclerite, and in the trifid lower sclerite, with two apically pointing spines similar in length.

This species is named to honor the American poet Joyce Kilmer “I think that I shall never see, a poem as lovely as a tree...” for whom the type locality stands as a proper monument to his appreciation for nature.

#### ﻿ Euconnus draco sp. nov.

Taxon classificationAnimaliaColeopteraStaphylinidae

277589C8-1691-5171-8249-3B2A2D722382

https://zoobank.org/4C7107D3-59AB-4C24-BBFF-A56EDD2568AB

Figs 11 , 12A

##### Type material.

***Holotype*** ♂, deposited in FMNH: “USA:NC: Graham Co., 35.3216°N, 83.9929°W, NantahalaNF, v.4.2020 5522’, Huckleberry Knob, M.Caterino & F.Etzler, sifted spruce litter” / “[QR code] CLEMSON-ENT CUAC000135352” / “Caterino DNA Voucher Extraction MSC6877 Morphosp.HKnb.B.329”. ***Paratypes*** (6, CUAC, FMNH) – 3 ♂: same data as type; 1 ♀: “USA:NC: Graham Co., 35.3210°N,83.9934°W, NantahalaNF, v.4.2020, 5491’, Huckleberry Knob, M.Caterino&F.Etzler, sifted deciduous litter” / “[QR code] CLEMSON-ENT CUAC000003924” / “Caterino DNA Voucher Extraction MSC4308 Morphosp.HKnb.A.048”; 1 ♀, 1 ♂: “USA:NC: Graham Co., 35.3171°N,83.9833°W, NantahalaNF, v.4.2020, 4702’, Cherohala Skyway, M.Caterino & F.Etzler, sifted deciduous litter”

##### Diagnostic description.

**Figure 12.**
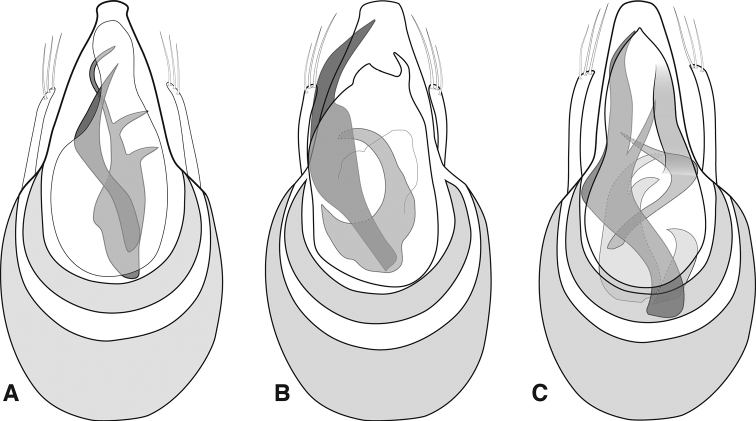
Aedeagus. **A***Euconnusdraco***B***Euconnustusquitee***C***Euconnusattritus*.

This species exhibits no obvious external differences from the preceding three, and can best be distinguished by male genitalic characters; its male antennomere IX has the apical corner quite dentate; aedeagus (Fig. 12A) with apex of median lobe narrowly knobbed and bluntly rounded; parameres weakly curving inward to apices, each bearing three long apical setae; compressor plate weakly asymmetrical, broadly rounded, nearly reaching to apex of median lobe; endophallus with two asymmetrical primary sclerites, upper sclerite long and narrowly sickle-shaped, its apex straight and acute; lower sclerite with three inwardly directed hooks, the apical-most long, thin, curved, median hook thicker, more strongly bent inward, basal-most thick, short, close to median.

##### Distribution.

*Euconnusdraco* is also basically a single site endemic, known only from the vicinity of Huckleberry Knob, at 5500ft in the Unicoi Mts. The second, Cherohala Skyway locality is only 1km away, and just slightly lower at 4700ft.

##### Remarks.

The long, trifid lower endophallic sclerite of *E.draco*, with the apical hook long and thin, best distinguishes this species. The shape of the median lobe, compressor plate, and upper endophallic sclerite are otherwise quite similar to *E.falcatus*.

This name of this species derives from the Latin ‘dragon’, as its type locality is near the popular motoring route ‘Tail of the Dragon’.

#### ﻿ Euconnus tusquitee sp. nov.

Taxon classificationAnimaliaColeopteraStaphylinidae

0267D10B-2FCC-5C1F-B46B-080972D9282F

https://zoobank.org/7F5099B6-92EE-4C41-B3FE-7C6203F5554D

Figs 11 , 12B

##### Type material.

***Holotype*** ♂, deposited in FMNH: “USA:NC: Clay Co., 35.1419°N, 83.7269°W, NantahalaNF, Tusquitee Bald, vii.6.2021, 5270’, *Quercus*/*Tsuga* litter M.Caterino & E.Recuero” / “[QR code] CLEMSON-ENT CUAC000171987” / “Caterino DNA Voucher Extraction MSC11727 Morphosp.TsqBld_171987”. ***Paratypes*** (1) – DNA only, voucher lost in extraction: same data as type, DNA Extract 7816, MorphospeciesTsqB.A.223.

##### Diagnostic description.

This species exhibits no obvious external differences from the preceding species, and can best be distinguished by male genitalic characters; carina of male antennomere VIII slightly oblique, ~ 20 degrees off the long axis of the antenna; aedeagus (Fig. 12B) with apex of median lobe narrowed to near truncate apex, not knobbed; parameres weakly curved, bearing three apical setae; compressor plate short, strongly asymmetrical, sinuate with blunt median and acute lateral lobes; endophallus with dominant upper asymmetrical sclerite long, broad to middle, with narrow, curving apical portion nearly reaching apex of median lobe, bearing an inner, medially directed secondary tooth just beyond middle; lower endophallic sclerite shorter, reaching just to middle of upper, curving strongly opposite.

##### Distribution.

Another single-site endemic, *Euconnustusquitee* is only known from Tusquitee Bald in the western Nantahala Mts, at an elevation of 5270 ft.

##### Remarks.

The strongly asymmetrical and unevenly bilobed apex of the compressor plate is the best character for recognizing *E.tusquitee*. The strongly opposing hook on the lower endophallic sclerite is also distinctive. Its sickle-shaped upper endophallic sclerite is longer than, but otherwise similar to that of *E.kilmeri*.

DNA of two specimens of this species was extracted. The first one was lost in the extraction process, though its DNA and images on the Flickr site (morphospecies code TsqB.A.223) remain. Unfortunately, neither extract sequenced well, so the placement of this species among the others of the *E.falcatus* complex remains uncertain.

This species name refers to its type and only known locality, Tusquitee Bald.

#### ﻿ Euconnus attritus sp. nov.

Taxon classificationAnimaliaColeopteraStaphylinidae

67BE608F-976A-50CE-B559-ADBB12F2355D

https://zoobank.org/37927727-6073-4A5B-8B1C-9281D47B3699

Figs 11 , 12C , 13

##### Type material.

***Holotype*** ♂, deposited in FMNH: “USA:NC: Mitchell Co., 36.0931°N, 82.1453°W, Roan High Bluff, 6225’ viii.15.2018, M.Caterino, sifted *Abies* litter” / “[QR code] CLEMSON-ENT CUAC000003146” / “Caterino DNA Voucher Extraction MSC3360 Morphosp.RHB.A.012”; ***Paratypes* (62**, CUAC, FMNH, CNCI, UNHC) – 3 ♀, same data as type; 18 ♀, 16 ♂: “USA:NC: Mitchell Co., 36.0933°N, 82.1447°W, Roan High Bluff, 6251’ viii.15.2018, M.Caterino, sifted *Abies* litter”; 1 ♀, 1 ♂: “USA:NC: Mitchell Co., 36.0999°N, 82.1345°W, Roan High Bluff, 6146’, viii.15.2018, M.Caterino, *Rhododendron* litter”; 2 ♀, 12 ♂: “USA: NC: Mitchell Co., 36.1041°N, 82.1223°W, PisgahNF, vi.8.2020, Roan High Knob, 6276’, M. Caterino, conifer litter”; 5 ♀, 4 ♂: “USA: NC: Mitchell Co., 36.1045°N, 82.1224°W, PisgahNF, vi.8.2020, Roan High Knob, 6286’, M. Caterino, conifer litter”.

##### Other material.

(52) – **TN**: Unicoi Co., Cherokee National Forest, Big Bald, 5237–5346ft, 5-Aug-2020 (1 ♀, 5 ♂); **NC**: Avery Co., Grandfather Mt., 5240–5370ft., 21-Apr-2022 (4 ♀, 5 ♂); Caldwell Co. Grandfather Mt. State Park, Calloway Peak, 6-Oct-2020 & 17-May-2021, 5775–5915ft. (12 ♀, 13 ♂); Caldwell Co. Grandfather Mt. State Park, Nuwati Tr., 4190ft. (1 ♂); Yancey Co., Pisgah National Forest, Woody Ridge Tr., 5086–5387ft., 15-Jun-2020 (5 ♀, 4 ♂); Yancey Co., Pisgah National Forest, Celo Knob, 6300ft., 19-Oct-2021 (1 ♂); Madison Co., Pisgah National Forest, Camp Creek Bald, 4741ft., 1-Mar-2022 (1 ♂).

##### Diagnostic description.

**Figure 13.**
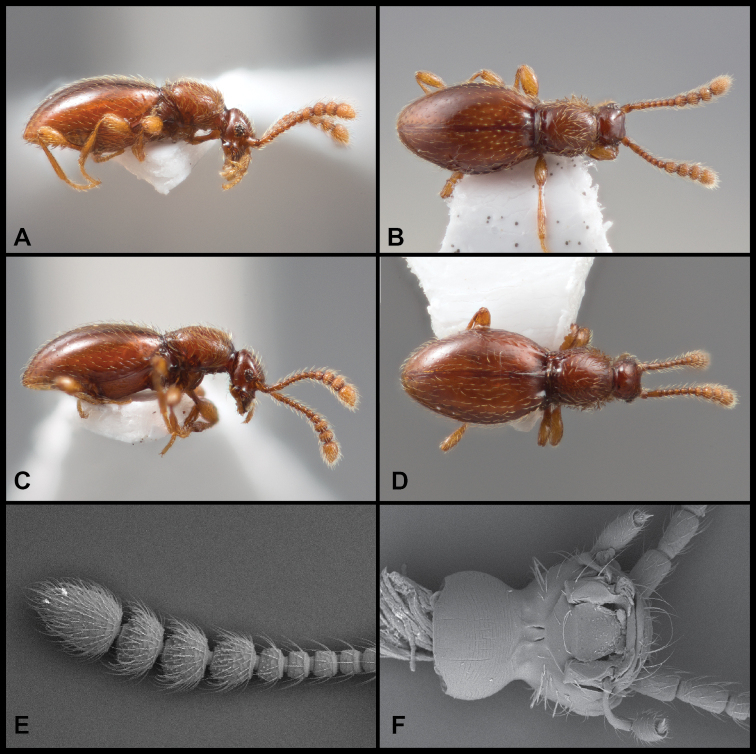
Habitus and character photos of *Euconnusattritus***A** male, lateral view **B** male, dorsal view **C** female, lateral view **D** female, dorsal view **E** SEM of male antenna **F** SEM of venter of head.

This species is extremely similar in external morphology to many of the preceding, and is also best distinguished by male genitalic characters. However, it does exhibit a few unusual characters. The male antennomeres VIII-IX are slightly enlarged, but lack carinae on their inner/anterior edges (Figs 13A, E); the female’s antennal club is shorter, and essentially trimerous (Fig. 13D), though antennomere VIII is slightly enlarged relative to VII; males and females flightless; median lobe of aedeagus (Fig. 12C) relatively long, nearly as long as basal bulb, narrowed to bluntly rounded, but not knobbed apex; shoulders of aedeagus sloped; parameres weakly curved, apices tapered, bearing three (rarely two) long straight setae; compressor plate symmetrical, parallel-sided to near apex, then narrowed to subacute apex, nearly as long as median lobe; endophallus with single long, upper sclerite, strongly sinuate, apically acute, bearing a sharp secondary process near its midpoint; the right side of the compressor plate sclerotized in a linear band, appearing as a second similar linear sclerite; actual second upper sclerite is much shorter, bent strongly at thickened midpoint, tapering to a thin, acute tip; lower endophallic sclerite a similar-length, trifid claw-like process on a sinuate stem originating near basal orifice.

##### Distribution.

This species is known only from northeast of the Asheville depression, although there it is moderately widespread, occurring in all the major ranges: the Black Mts, Roan Highlands, Grandfather Mountain, Big Bald, and Camp Creek Bald. Its known localities span an elevation range of 3800–6300 ft. There is a CUAC specimen labelled ‘Toxaway Mountain’ (in the more southerly Nantahala Mts.) that is almost certainly mislabeled, as that site was visited the same day as the Big Bald locality.

##### Remarks.

Of the pale species of *Cladoconnus*, this species is only sympatric with *E.adversus* (it is also sympatric with the larger and darker *E.vetustus* and *E.vexillus*). Males of *E.adversus* have very conspicuous antennal carinae, which are completely lacking in *E.attritus*. Females, however, will be indistinguishable. Males of *E.attritus* have very distinctive endophallic sclerites, particularly the long, sinuate left upper sclerite, with its secondary median spike.

The name of this species suggests that the subgenus-typical male antennal carinae are ‘worn away’.

#### ﻿ Euconnus adversus sp. nov.

Taxon classificationAnimaliaColeopteraStaphylinidae

6CD9C9BD-FB24-525C-AE52-26ACDE0AB23C

https://zoobank.org/E8D7FF15-E9B6-48EE-9191-E1C4E7ABE41B

**Figure 14.**
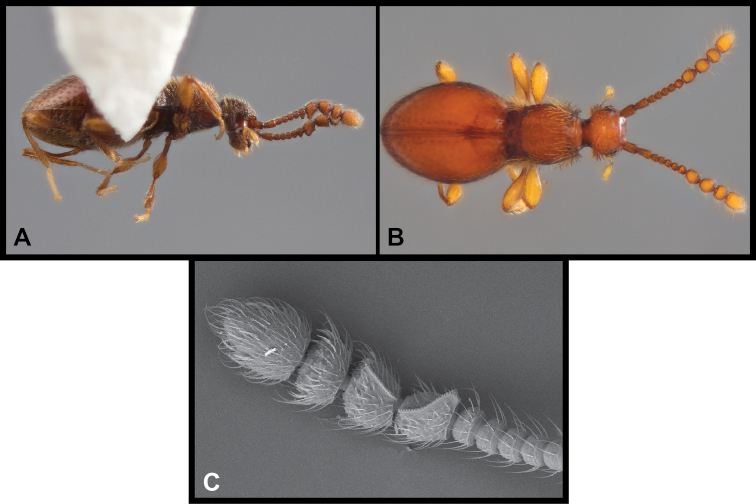
Habitus and character photos of *Euconnusadversus***A** male, lateral view **B** male, dorsal view (NT.A.009) **C** SEM of male antenna.

**Figure 15.**
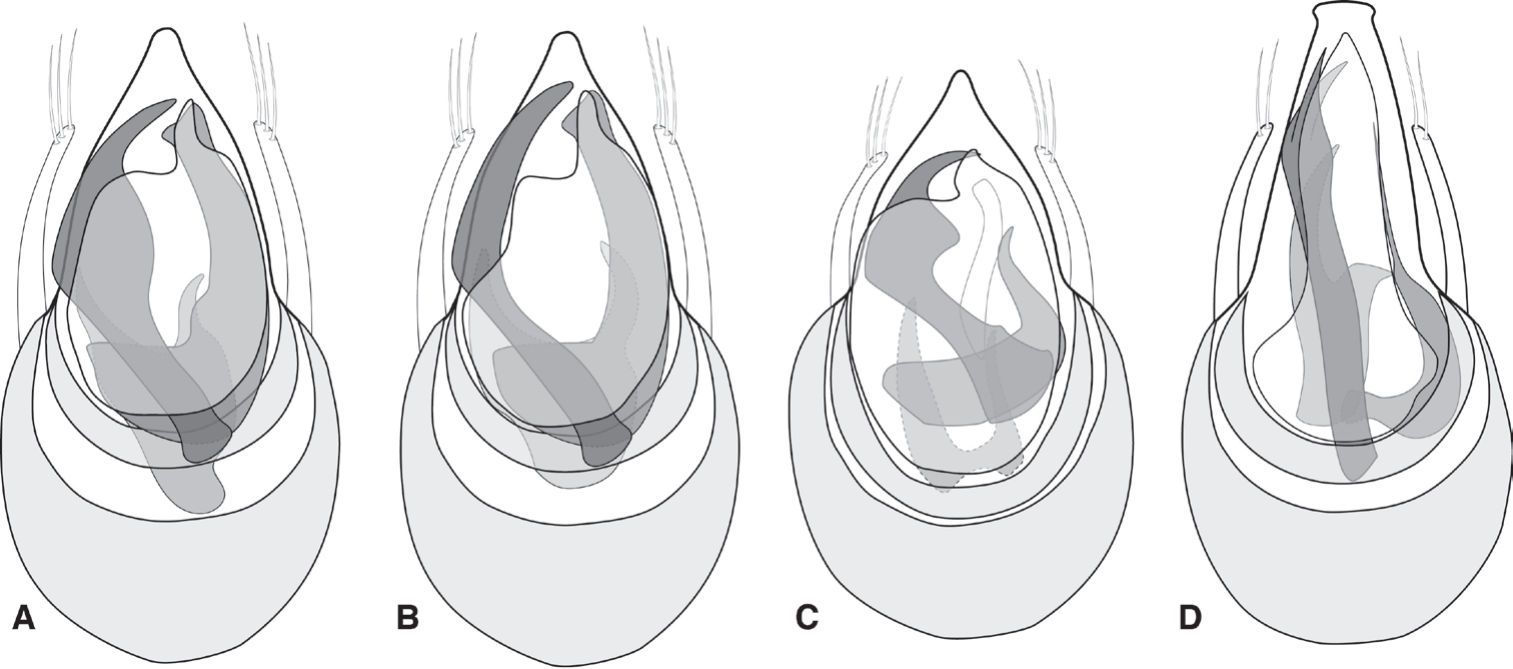
Aedeagus **A***Euconnusadversus* (type locality – Celo Knob, NC) **B***Euconnusadversus* (non-type locality – Chestnut Ridge, SC) **C***Euconnusastrus***D***Euconnuscultellus*.

Figs 11 , 14 , 15A, B

##### Type material.

***Holotype*** ♂, deposited in FMNH: “USA: NC: Yancey Co., 35.8524°N, 82.2485°W, PisgahNF, CeloKnob, x.19.2021, 6300’, M.Caterino, E.Recuero, & A.Haberski, sifted litter” / “[QR code] CLEMSON-ENT CUAC000157519” / “Caterino DNA Voucher Extraction MSC9225 Morphosp.CK.B.370”. ***Paratypes*** (4, CUAC) – 1 ♂: “USA: NC: Yancey Co., 35.8447°N, 82.2369°W, PisgahNF, Woody Ridge Tr., vi.15.2020, 5244’, M.Caterino, F.Etzler, sifted litter” / “[QR code] CLEMSON-ENT CUAC000137633” / “Caterino DNA Voucher Extraction MSC5528 Morphosp.WR.A.046”; 1 ♂: “USA: NC: Caldwell Co., 36.1184°N, 81.7909°W, Grandfather Mt.SP, 4020’, Nuwati Tr., v.17.2021, A.Haberski, P. Wooden, sifted litter” / “[QR code] CLEMSON-ENT CUAC000135057” / “Caterino DNA Voucher Extraction MSC6364 Morphosp.NT.A.009”; 1 ♂: “USA: NC: Buncombe Co.Co. 35.7955°N, 82.3392°W, Big Butt Tr.,iii.19.2016, S.Myers, L.Vasquez-Velez, sifted litter” / “[QR code] CLEMSON-ENT CUAC00026846”; 1 ♂: “USA: TN: Unicoi Co., 35.9950°N, 82.48972°W, Cherokee NF, Big Bald, v.21.2021, CW. Harden, A.Haberski, P.Wooden, sifted litter” / “[QR code] CLEMSON-ENT CUAC000135395” / “Caterino DNA Voucher Extraction MSC6920 Morphosp.BgBld.A.026”.

##### Other material.

(19): **NC**: Jackson Co., Balsam Mountain Preserve, Sugarloaf Mountain 4491ft, 7-Feb-2015, Sifting litter, oak litter in old stump depression; Ashe Co., Mt. Jefferson State Park, SE Reservoir, 4-Jul-1960; **SC**: Greenville Co., Chestnut Ridge Heritage Preserve, 1140 & 1220 ft., 5-June-2015; Pickens Co., Clemson Experimental Forest, Seed Orchard Rd., 700ft, 12-Jul-2016; Oconee Co., Ellicott Rock Wilderness, East Fork Chattooga River, 2110ft, 4-May-2015; Oconee Co., Ellicott Rock Wilderness, Indian Camp Creek, 2822ft, 4-May-2015; Greenville Co., Mtn. Bridges Wilderness, 2230ft., 10-Mar-2018; **GA**: Rabun Co., NE Pine Mt., Chattooga R., 1800ft, 5-Jun-1981 (CUAC, CNCI, FMNH).

##### Diagnostic description.

This species is generally very similar to the preceding, and can best be distinguished by male genitalic characters; it and the following, however, exhibit the most prominent antennal carinae among American *Cladoconnus*, those on antennomeres VIII and IX both being strong and oblique (Fig. 14C), that of VIII most produced at base and that of IX most produced at apex; antennomere VII also exhibits some expansion along its inner margin; males may be wing-polymorphic, winged individuals appearing larger and darker in body color; female wings not observed; aedeagus (Figs 15A, B) with median lobe evenly tapered and narrowly rounded at apex; parameres thin, each bearing three short terminal setae, the setae not reaching apex of median lobe; compressor plate short, asymmetrical, truncate on one side (‘left’ as drawn), produced on other; upper endophallic armature comprising two dominant curved sclerites, one tapered to narrowly subacute apex, the shorter one much broader, more weakly curved inward to a bluntly truncate apex that meets the apex of the other; the lower endophallic armature consisting of a shorter, deeply bifurcate (or trifurcate) process, the apices slightly varied in curvature, generally directed distad.

##### Distribution.

This species is widespread but rare, found at scattered lower elevation sites across northwestern South Carolina, to higher elevations in the Nantahalas, Blacks, Grandfather Mountain, and Mount Jefferson in northernmost North Carolina. It has a broad elevational range as well, from just 700 ft up to the highest peaks in the region at 6500 ft. A single male labelled as from the Florida panhandle is almost certainly mislabeled. Collected by Stewart Peck on 8 June 1981, it was collected just 3 days after he collected another specimen of this species in Rabun County, Georgia. The ‘Florida’ specimen probably belongs to the Georgia series.

##### Remarks.

The strongly modified antennomeres of male *E.adversus* will distinguish them immediately from anything sympatric (though not the more western *E.astrus*, below). There is considerable variation site-to-site in the detailed shapes of the upper and lower endophallic armature. In males from the Balsam Mt. Preserve (NC), the innermost endophallic sclerite is deeply trifucate, whereas in those from the Chestnut Ridge Heritage Preserve (SC) the right tip of the lower endophallic sclerite is curved inward (compare Fig. 15B and Fig. 15A, respectively). None of these southern localities, however, are represented by sequence data, so before recognizing these variants taxonomically, better representation for molecular data would be advisable.

The name of this species refers to the seemingly ‘opposable’ carinae of male antennomeres VIII and IX.

#### ﻿ Euconnus astrus sp. nov.

Taxon classificationAnimaliaColeopteraStaphylinidae

63051B25-B201-5FD3-9631-4B9496F37A0A

https://zoobank.org/D02BE2CE-A8EE-44C8-995F-5C69F95FB117

Figs 7 , 15C

##### Type material.

***Holotype*** ♂, deposited in CMNC: “ALA., Jackson Co., 5mi.N.W.Princeton, 19.V.1972, S.Peck. Ber.240” / “Caterino DNA Voucher Extraction MSC12286 Cladoconnus(AL)”; ***Paratypes*** (3): 1 ♂, same data as type; (FMNH); 1 ♂, 1 ♀: “nr. Jess Elliot Cave, Jackson Co., ALA. 8.IV.1961” / “Hollow Tree, V.D.Patrick, H.R.Steeves, leg.”

##### Other material.

(4) – **AL**: Marshall Co., Grant, 25-May-1958 (1 ♂); Colbert Co., Maud, nr. McCluskey Cave, 26-Mar-1962 (3 ♀, 1 ♂).

##### Diagnostic description.

This species is externally identical to *E.adversus*, above, and can only be distinguished by male genitalic characters. Both species share very prominent antennal carinae (e.g., Fig. 14C), those on antennomeres VIII and IX both strong and oblique, that of VIII most produced at base and that of IX most produced at apex. They similarly appear to be wing-dimorphic, with winged males and wingless females; aedeagus (Fig. 15C) with median lobe evenly tapered and narrowly rounded at apex; parameres thin, each bearing three short terminal setae, the setae not reaching apex of median lobe; compressor plate short, asymmetrical, truncate on one side (left as drawn), narrowly and unevenly produced on other; left upper endophallic sclerite strongly hooked, with prominent, blunt median tooth on inner margin; right upper endophallic sclerite a short curved spine, the tip pointing laterad; lower endophallic armature comprising a deeply bifurcate process, with two long, slightly sinuate spikes pointed distad.

##### Distribution.

This species is only definitely known from northeastern Alabama, where it has been found in a few caves, as well as a few free-living situations. A series from Colbert County, in northwestern Alabama, comprises only females. Males from this locality would be interesting to examine.

##### Remarks.

This species appears closely related to *E.adversus*, but differs substantially in genitalic characters, with the right upper endophallic sclerite quite different, and the lower endophallic sclerites much more elongate. The species name means ‘starry’, referring to the nearby NASA rocket science and spacecamp facilities.

#### ﻿ Euconnus cultellus sp. nov.

Taxon classificationAnimaliaColeopteraStaphylinidae

901E5EC7-DB12-5C50-BFF5-39DBAB25FEEB

https://zoobank.org/796C1879-2BB4-4515-815D-FDBDAF6C43AD

Figs 15D , 16 , 17

##### Type material.

***Holotype*** ♂, deposited in FMNH: “USA: GA: Rabun Co., 34.9658°N, 83.2997°W, ChattahoocheeNF, Rabun Bald, 4663’, v.11.2021, sifted litter, M.Caterino & A.Haberski” / “[QR code] CLEMSON-ENT CUAC000146083” / “Caterino DNA Voucher Extraction MSC12039”. ***Paratypes*** (8) – 5 ♀, 5 ♂: same data as type.

##### Other material.

(28) **GA**: Clay Co., Chattahoochee National Forest, Brasstown Bald, 4590ft, 19-Sep-2015 (3 ♂); **NC**: Cherokee Co., Nantahala National Forest, Hickory Branch trail, 3923ft., 26-Jul-2015 (1 ♂); Cherokee Co., London Bald Tr., 4108ft, 26-Jul-2015 (2 ♀); Graham Co., Nantahala National Forest, Teyahalee Bald, 4591ft., 12-Apr-2022 (1 ♂); Macon Co., Nantahala National Forest, Cowee Bald, 4942ft., 9-Jul-2019 (2 ♂); Macon Co., Nantahala National Forest, E. Highlands, Hwy 64, 3880ft., 1-Mar-2020 (1 ♀ 1 ♂); Macon Co., Nantahala National Forest, Copper Ridge Bald, 5032ft., 15-Sep-2020 (1 ♂); Swain Co., Nantahala National Forest, Miller Cove app trail, 2323ft., 20-Jul-2015 (2 ♀, 6 ♂); **SC**: Oconee Co., Buzzards Roost Heritage Preserve, 1250ft., 16-Jan-2015 (1 ♂); Oconee Co., Chau-Ram Country Park, 850ft., 15-Oct-2015 (1 ♀, 1 ♂); Oconee Co., Sumter National Forest, Yellow Branch Falls, 1560ft., 12-Oct-2017 (2 ♀, 1 ♂); Oconee Co., Sumter National Forest, Chattooga river, 1580ft., 2-Apr-2015 (1 ♂); Clay Co., Nantahala National Forest, Tusquitee Bald, 5015ft., 1-Sep-2020 (1 ♂).

##### Diagnostic description.

**Figure 16.**
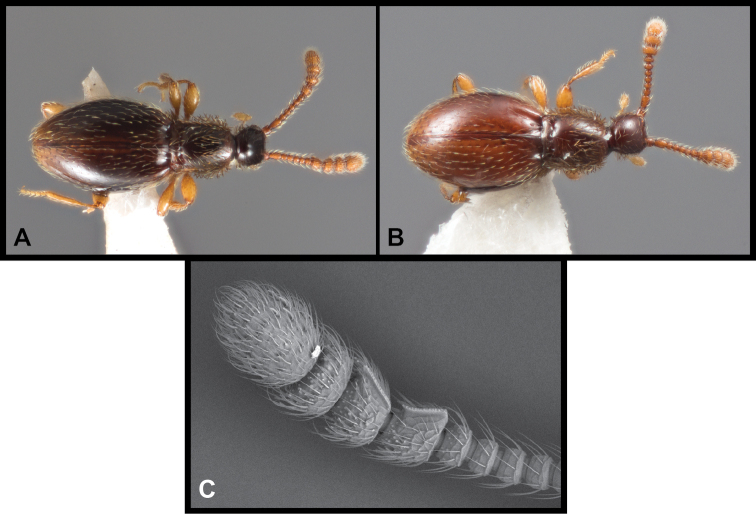
Habitus and character photos of *Euconnuscultellus***A** male, dorsal view **B** female, dorsal view **C** SEM of male antenna.

This species exhibits few obvious external differences from the preceding ‘*falcatus* complex’, and can best be distinguished by male genitalic characters; body color sometimes darker (Fig. 16A); carina of male antennomere VIII (Fig. 16C) more strongly inclined, produced further at the base, than in most of the previous (except *E.adversus* and *E.astrus*), and carina of male antennomere IX slightly oblique, ~ 20 degrees off the long axis of the antenna; males at least sometimes winged, females apparently wingless; aedeagus (Fig. 15D) with apex of median lobe knobbed, bluntly truncate; parameres short, curved, bearing 3 setae at their apices; compressor plate more or less symmetrical, narrow, parallel-sided from near base, sinuately tapering to subacute apex; endophallic armature with upper blade long, nearly straight, widened beyond middle, curving and tapered to bifid, acute apices (a second tooth projecting straight behind the uppermost); a second upper sclerite bends laterad strongly from the base, curving distad and tapering to long thin apex beneath ‘right’ edge of compressor plate; lower sclerite with two strong hooks, the basalmost broad and blunt, the apicalmost sinuately curving behind apex of upper endophallic sclerite.

**Figure 17.**
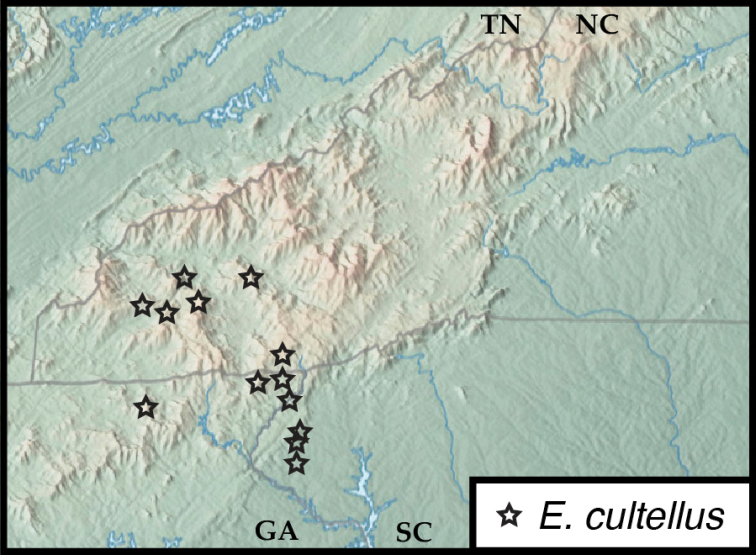
Map of collecting records for Euconnus (Cladoconnus) cultellus.

##### Distribution.

*Euconnuscultellus* occurs across a relatively limited portion of western North Carolina, in the Cowee and Nantahala Mts, northeast Georgia, and upstate South Carolina. Its distribution seems to be limited on the east by the Little Tennessee River system, not (yet) found east of the Tuckasegee tributary, and not having been found in the Great Smoky Mountains, or on any of the spruce-fir peaks sampled. That limit aside, it has a broad elevational range, occurring from 850–5000ft.

##### Remarks.

The bifid apex of the upper endophallic sclerite is distinct from all other *Cladoconnus* species except *E.cataloochee*. The distinctive broad basal hook of the lower endophallic sclerite differentiates *E.cultellus* from all others, as does the deeply curved, apically slender, tapering right arm of the upper armature.

This species is named for the ‘cutting’ edge of the males finely serrate antennal carinae.

### ﻿Phylogeny

**Figure 18.**
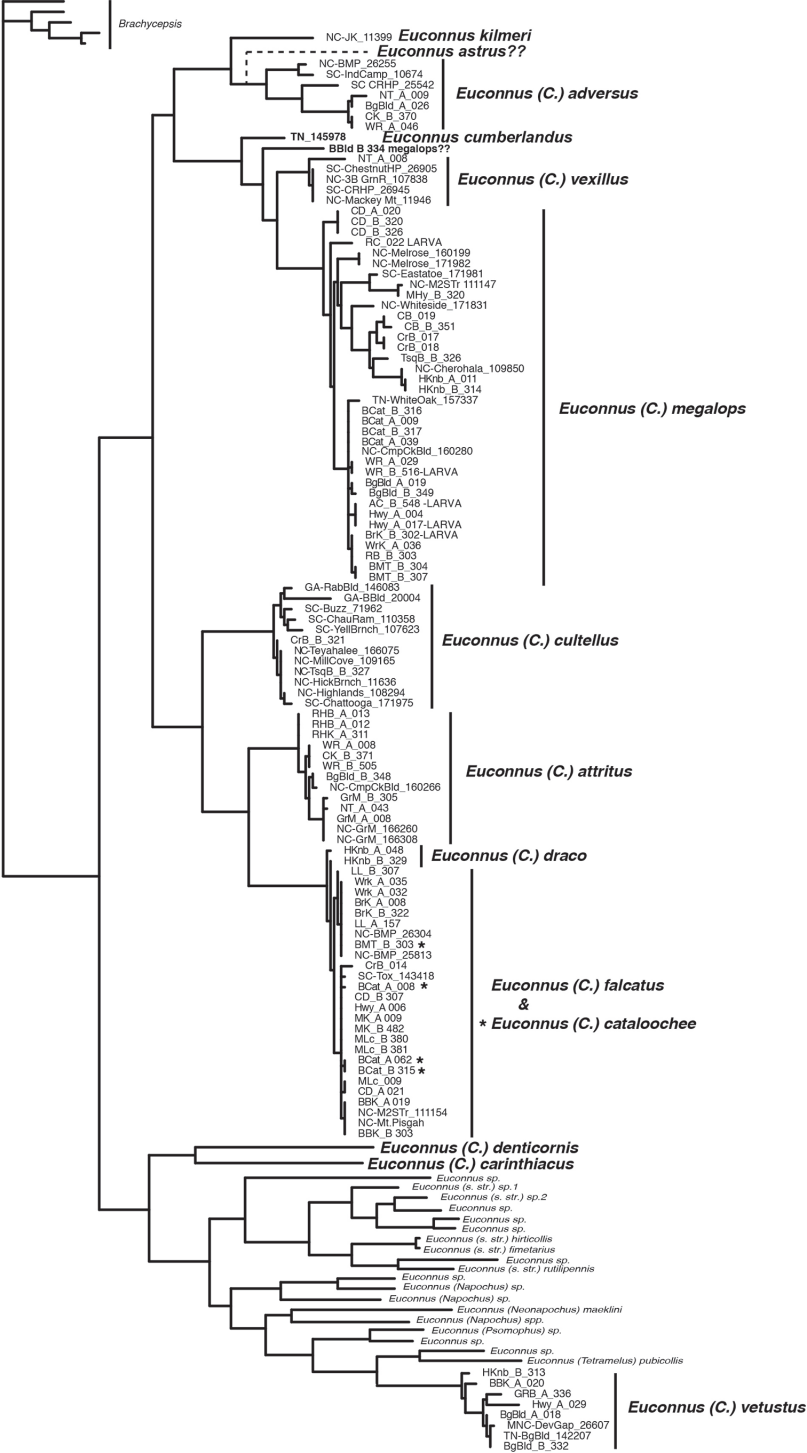
Phylogeny of American species of Euconnus (Cladoconnus). Hypothesized position of *E.astrus*, for which no sequence data is available, is indicated with a dotted line.

Phylogenetic analysis of available COI sequences does not support monophyly of American *Cladoconnus* relative to all other *Euconnus* (Fig. 18). Most of the new species fall out in one large clade. But *Euconnusvetustus* is resolved as a separate clade outside all the others. This is a dark, flightless species that lacks male antennal modifications. It is also an unusually widespread species, known from several localities on both sides of the Asheville depression. Neither lineage falls out near the two European *Cladoconnus*, E. (C.) denticornis Müller & Kunze and E. (C.) carinthiacus Ganglbauer for which COI sequences are available, with those forming a sister lineage to most of the rest of *Euconnus* (within which the *Cladoconnusvetustus* lineage falls out). However, it must be acknowledged that all these lineages (the three distinct ‘*Cladoconnus*’ lineages) are highly divergent (> 20% K2P distance), and COI struggles to resolve these deeper relationships. It is worth noting that two American *Euconnussensu stricto* species fall out near some European members of that subgenus, and far from any of the newly described *Cladoconnus* – these new species had initially been considered as potentially belonging there. Overall, these deeper results are based on a very sparse sampling of global *Euconnus* diversity, and only a small fragment of a single mitochondrial gene, so can only be given so much credence.

Within the main American *Cladoconnus* lineage, the species are divided into two main clades, one containing *Euconnusmegalops*, *E.cumberlandus*, *E.vexillus*, *E.adversus*, and *E.kilmeri*. *Euconnusastrus* is not represented by molecular data, but it may be expected to be a member of this group, likely as sister to *E.adversus*. *Euconnusmegalops* is probably not yet adequately resolved, as one sample considered to belong there (the Brasstown Bald locality) falls well outside the main group, as sister to a *Euconnusmegalops* + *E.vexillus* lineage. But this male’s genitalia do not differ obviously from the rest of *E.megalops. Euconnusvexillus*, on the other hand, exhibits distinct male genitalia and male antennal characters, so is clearly a distinct species. More sequences and more genitalia from outlying localities will be necessary to resolving this uncertainty. There is also considerable genetic diversity within the widespread species *E.megalops*. But it shows relatively little geographic structure, with populations from both sides of the Asheville Basin somewhat intermingled. There is one subclade within this that shows a higher degree of genetic variation, from mostly southwestern localities (Huckleberry Knob, Tusquitee Bald, etc.). But these show no obvious morphological coherence.

The other large clade has *Euconnuscultellus* as sister to *Euconnusattritus* and several very closely related species of the *Euconnusfalcatus* complex. *Euconnuscultellus* occurs in only the southern part of the region, from scattered localities in the Nantahala Mountains just into far western South Carolina. There is considerable genitalic variation site-to-site, but their sequences, while showing some diversity, don’t suggest significant differentiation. *Euconnusattritus* occurs only in the northeastern part of the region, including the Blacks, Grandfather Mt., and the Roan Highlands, showing potentially meaningful COI differences among the localities. The *Euconnusfalcatus* complex, on the other hand, includes three quite distinctive genitalic forms that show surprisingly little COI differentiation. One, *Euconnuscataloochee*, is even scattered within another both genetically and geographically (they are marked by asterisks in Fig. 18). This non-monophyly is peculiar and requires further genetic data to resolve. The genitalic differences between *E.falcatus* and *E.cataloochee* are marked and consistent. Two individuals representing *E.draco* resolve as sisters to the remainder of this clade, but are not themselves monophyletic (despite coming from the same locality, Huckleberry Knob). *Euconnustusquitee* would also be expected to be very similar to these, but it has not yet yielded clean sequence data. These results together indicate either rapid speciation, over a time frame insufficient for coalescence of species-specific haplotypes, or a significant slow-down in mitochondrial evolution. Again, additional markers will be necessary to resolve this question.

## ﻿Discussion

The discovery of a diverse, previously unreported radiation of beetles in the southern Appalachians is surprising, even considering their small size and cryptic habits. The region has been popular with collectors and researchers for many years, and while new species are still encountered commonly, it is rare for major lineages to have escaped detection. Scydmaeninae have perhaps received less attention from taxonomists over the years than more prominent groups, and one could imagine other ‘hidden’ radiations in other similarly neglected arthropod taxa.

While only European members of *Cladoconnus* have previously been sequenced, it is worth considering whether the relationships of the Appalachian species lie among the Asian species. There aren’t any particular morphological characters that suggest such a relationship. But the existence in Japan and Korea of species that are sexually dimorphic in the possession of flight wings, as observed in several Appalachian species, might be informative. Then again, that begs the question of how a lineage with flightless females may have reached the area to begin with. But the deep genetic divergences, and somewhat incongruous distributions of some of the species (in particular, *E.vetustus* ignoring the biogeographically significant Asheville Depression) together suggest that *Cladoconnus* has been resident in the southeastern US for a very long time. Given that, however, it is surprising that their overall distribution is still somewhat limited. The species found to the west, in parts of the Cumberland Plateau, extend somewhat beyond Appalachia proper. But none have yet been found to occur in higher elevations of Virginia. Some older lineages of Appalachian arthropods find relatives in the Ouachita Mts of Arkansas (flightless *Lathrobium*, for example – Watrous and Haberski, pers. comm.; *Arianops* Brendel – Carlton 1990; *Anillinus* Casey – Allen 1990, Carlton and Robison 1998). It might be worth examining such collections more closely for overlooked representatives.

**Figure 19.**
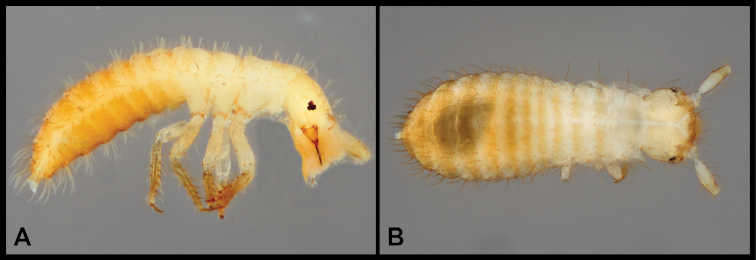
Larvae of Euconnus (Cladoconnus) megalops**A** dorsal view (specimen from Rabun Cliffs, GA) **B** lateral view (specimen from Woody Ridge Trail, NC).

Our ‘barcode-everything’ approach revealed several larvae of *Cladoconnus*, all, as definitely associated by DNA placement, of E. (C.) megalops (Fig. 19). Of the seven extracted and sequenced, vouchers of six survived the process more or less intact. No larvae of the larger genus *Euconnus* have yet been formally described (Newton 1991; Jałoszyński. pers. comm,), and I do not do so here. However, these represent a potentially valuable source for future character data for reconstructing scydmaenine relationships. Generally, the larvae resemble other described Glandulariini, such as *Stenichnus* Thomson (Wheeler and Pakaluk 1983; Newton 1991; Jałoszyński and Kilian 2012), prognathous, elongate, setose, slightly flattened, with a rather broad abdomen, and a large antennomere II with a simple, domelike apical cap.

**Figure 20.**
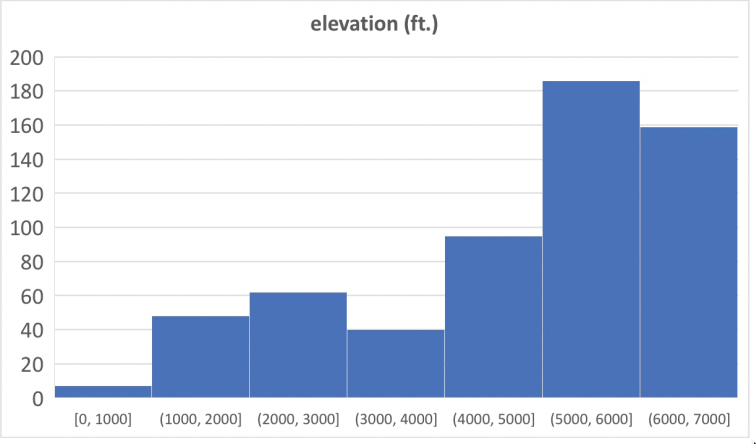
Histogram of record counts (y-axis) by elevation (x-axis, range in feet).

While the bulk of our records seem to suggest an exclusively high-elevation restricted group, with more than two-thirds of the available records coming from above 4500 feet (Fig. 20), the ranges of many of the species extend considerably lower, even below 1000 feet. These lower records tend to be from deep riparian canyons, and the higher humidity of such locations is probably part of the explanation. However, there is also a clear sampling bias here, in that recent fieldwork by our lab has targeted high elevation sites. Still, scanning our own older, lower elevation samples, and attempting to borrow specimens from other collections where lower elevation samples were better represented turned up relatively few additional specimens. It may be that many of these beetles have been ignored or overlooked in the abundant morass of litter microcoleoptera. But there have been a number of dedicated devotees over the years, including Sean O’Keefe, Donald Chandler, Christopher Carlton, Walter Suter, Thomas Barr, and others, so the apparent predilection of *Cladoconnus* for higher elevations seems likely to be real.

**Figure 21.**
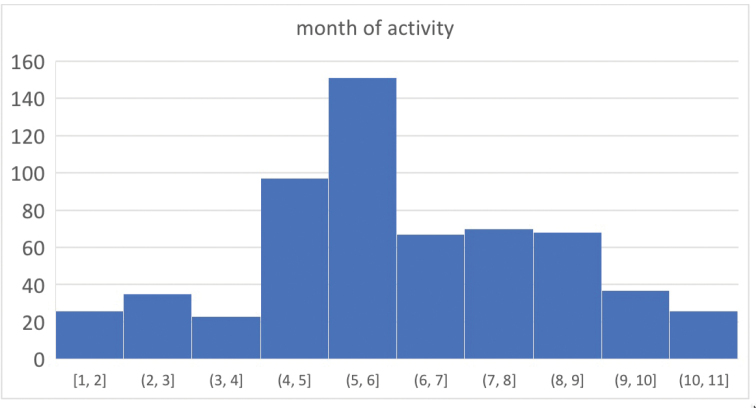
Histogram of record counts (y-axis) by month (x-axis).

Activity patterns of the species present similar questions. The majority of records come from late spring and early summer samples (Fig. 21), and such a pattern would conform to many species in the region. Our own sampling has had some of this bias, although for the past several years, during which most of these beetles were collected, similar emphasis was given to fall sampling, in September and October, and there is no indication of a secondary peak of specimen records, so there is probably some meaningful signal there, as well.

The range in genitalic diversity, especially among the apparently closely related species in the *E.falcatus* complex, is remarkable. In those species known from multiple localities, it seems to be the rule that variation in specific conformation of endophallic sclerites can be seen. I’ve taken a generally conservative approach in considering many to be widespread, variable species for now. But closer study could well split some of these more finely.

It would be fascinating to understand the *in situ* mechanisms of these species incredibly complex genitalia. It is tempting to hypothesize explanations of sperm competition and perhaps rival sperm removal, or of active female choice spurring an arms-race of male elaborations for maintaining hold and proper position. The dimorphic antennomeres of at least some of these species might also point to some similar sorts of intersexual dynamics. Unfortunately, for the present such a discussion would be pure speculation. Simply understanding the physical mechanisms that deploy the endophallic sclerites during intromission would constitute a major undertaking. Yet it might repay the effort, as similar dynamics may pertain to the diversification of a wide variety of ‘dark taxa’, within Staphylinoidea at least, where such structures are commonplace. For now, it must suffice to call attention to these remarkable creatures, and hope that future workers with a more applicable skillset take up the challenge.

## Supplementary Material

XML Treatment for Cladoconnus

XML Treatment for Euconnus (Cladoconnus) megalops

XML Treatment for Euconnus vexillus

XML Treatment for Euconnus cumberlandus

XML Treatment for Euconnus vetustus

XML Treatment for Euconnus falcatus

XML Treatment for Euconnus cataloochee

XML Treatment for Euconnus kilmeri

XML Treatment for Euconnus draco

XML Treatment for Euconnus tusquitee

XML Treatment for Euconnus attritus

XML Treatment for Euconnus adversus

XML Treatment for Euconnus astrus

XML Treatment for Euconnus cultellus

## Supplementary materials

Supplementary material 1 Specimen data for all material examined Data type occurrence Explanation note Specimen level data for all *Cladoconnus* specimens examined in this paper. Columns include type status, repository, voucher numbers (for Clemson University Arthropod Collection), morphospecies designation (which can be searched on Flickr for additional images), DNA extraction number (extractions in Caterino Lab/CUAC collection), GenBank accession numbers, and collection event and geographical information. https://binary.pensoft.net/file/786433This dataset is made available under the Open Database License (http://opendatacommons.org/licenses/odbl/1.0/). The Open Database License (ODbL) is a license agreement intended to allow users to freely share, modify, and use this Dataset while maintaining this same freedom for others, provided that the original source and author(s) are credited.Michael S. Caterino

Supplementary material 2 Phylogenetic character data (COI barcoding region) Data type DNA sequence Explanation note Nexus file containing all sequences analyzed in this paper, including Brachycepsis outgroups, new *Cladoconnus* species, new sequences for Nearctic Euconnus (Napochus) and *Euconnus* s. str. spp., and previously published *Euconnus* sequences from GenBank and BOLD. Line names contain their BOLD and/or GenBank Accession numbers for previously published sequences. https://binary.pensoft.net/file/786445This dataset is made available under the Open Database License (http://opendatacommons.org/licenses/odbl/1.0/). The Open Database License (ODbL) is a license agreement intended to allow users to freely share, modify, and use this Dataset while maintaining this same freedom for others, provided that the original source and author(s) are credited.Michael S. Caterino
